# COVID-19 Virulence in Aged Patients Might Be Impacted by the Host Cellular MicroRNAs Abundance/Profile

**DOI:** 10.14336/AD.2020.0428

**Published:** 2020-04-28

**Authors:** Sadanand Fulzele, Bikash Sahay, Ibrahim Yusufu, Tae Jin Lee, Ashok Sharma, Ravindra Kolhe, Carlos M Isales

**Affiliations:** ^1^Department of Medicine, Augusta University, Augusta, GA, USA.; ^2^Center for Healthy Aging, Augusta University, Augusta, GA, USA.; ^3^Department of Infectious Diseases and Immunology, University of Florida, Gainesville, FL, USA.; ^4^Center for Biotechnology and Genomic Medicine, Augusta University, Augusta, GA 30912, USA.; ^5^Departments of Pathology, Augusta University, Augusta, GA 30912, USA

**Keywords:** Coronavirus, microRNAs, aging

## Abstract

The World health organization (WHO) declared Coronavirus disease 2019 (COVID-19) a global pandemic and a severe public health crisis. Drastic measures to combat COVID-19 are warranted due to its contagiousness and higher mortality rates, specifically in the aged patient population. At the current stage, due to the lack of effective treatment strategies for COVID-19 innovative approaches need to be considered. It is well known that host cellular miRNAs can directly target both viral 3'UTR and coding region of the viral genome to induce the antiviral effect. In this study, we did *in silico* analysis of human miRNAs targeting SARS (4 isolates) and COVID-19 (29 recent isolates from different regions) genome and correlated our findings with aging and underlying conditions. We found 848 common miRNAs targeting the SARS genome and 873 common microRNAs targeting the COVID-19 genome. Out of a total of 848 miRNAs from SARS, only 558 commonly present in all COVID-19 isolates. Interestingly, 315 miRNAs are unique for COVID-19 isolates and 290 miRNAs unique to SARS. We also noted that out of 29 COVID-19 isolates, 19 isolates have identical miRNA targets. The COVID-19 isolates, Netherland (EPI_ISL_422601), Australia (EPI_ISL_413214), and Wuhan (EPI_ISL_403931) showed six, four, and four unique miRNAs targets, respectively. Furthermore, GO, and KEGG pathway analysis showed that COVID-19 targeting human miRNAs involved in various age-related signaling and diseases. Recent studies also suggested that some of the human miRNAs targeting COVID-19 decreased with aging and underlying conditions. GO and KEGG identified impaired signaling pathway may be due to low abundance miRNA which might be one of the contributing factors for the increasing severity and mortality in aged individuals and with other underlying conditions. Further, *in vitro* and *in vivo* studies are needed to validate some of these targets and identify potential therapeutic targets.

Novel Coronavirus was identified near the end of 2019, presenting with a spectrum of symptoms including febrile illness, cough, severe pneumonia, and in some patients, death. The pathogen is now widely called Coronavirus disease 2019 (COVID-19) and has rapidly turned into a global pandemic since originally being identified in Wuhan, China. Coronaviridae is the family of single-stranded (+ssRNA), pleomorphic, enveloped RNA viruses that consists of the Coronavirus genus [1]. COVID-19 is also referred to as severe acute respiratory syndrome coronavirus 2 (SARS-CoV-2) due to the genomic and symptomatic similarities with the SARS-CoV that caused an epidemic in 2002-2004 [2]. SARS-CoV-2 is predominantly spread by person-to-person contact via virally loaded respiratory droplets [3]. However, the transmission may also occur by touching contaminated surfaces and subsequently touching one's eyes, nose, or mouth [4, 5]. The incubation period, the period before the first symptoms present itself, is on average of 4-5 days, with 95% presenting within 12.5 days [6,7].

The World Health Organization (WHO) reported 2,920,905 confirmed infected, and 203,269 COVID-19 related deaths globally as of April 25th, 2020 [8]. Drastic measures to combat SARS-CoV-2 are warranted due to its contagiousness, viability, and higher death toll compared to its predecessor SARS-CoV. Although SARS-CoV had a significantly higher case-fatality (around 10%) [9], the virus was not viable enough to remain in the human population and never spread in the United States and other countries like SARS-CoV-2 [10]. SARS-CoV-2, on the other hand, appears to rapidly spread, which is contributing to its global spread and a significantly higher number of cases.

Severe and critical cases of COVID-19 disproportionately affect middle-aged and older/aged populations, with increased mortality in aged adults [11]. Illness severity ranges from asymptomatic, mild, severe, and critical. Mild illness is characterized as no to mild pneumonia [11]. The severe disease presents with dyspnea, hypoxia, or >50% lung involvement, and critical illness occurs when there is respiratory failure, shock, or multiorgan failure [11]. The Centers for Disease Control and Prevention (CDC) and the WHO estimate the mortality rates of SARS-CoV-2 to be around 3.4% and case fatality rates (CFR) may increase up to 10-27 % in individuals 80 years old or older [8, 12]. SARS-CoV-2 is a novel virus, and very little is known about it. In such a scenario, it is crucial to understand the COVID-19 pathobiology to identify the innovative treatment strategy. Our laboratory and others have reported that miRNAs play a critical role in age-related complications [13-16].

MiRNAs play a vital role in the pathogenesis of various diseases, including viral infections, disease progression, and inhibition [17-23]. MicroRNAs are small noncoding RNAs, bind to 3'UTR of mRNA, and inhibit translation or induce degradation of mRNAs [19, 21, 23]. Recent studies suggested that host cellular miRNAs can directly target both viral 3’UTR and coding region of the viral genome to induce antiviral effect [19, 21, 23]. For example, the number of groups previously reported that host miRNAs (miR-323, miR-491, miR-485, miR-654, and miR-3145) bind to influenza PB1 gene coding region, degrade RNA and inhibit viral translation and reduce the accumulation of viral particles [19, 24, 25]. Furthermore, the host cellular miRNA-29a inhibit Human immunodeficiency virus type 1 (HIV-1) nef protein expression and thus, inhibit viral replication [26]. On the contrary, some groups also suggested the positive effect of host miRNAs on viral replication. For example, miR-122 binding to 3’ and 5’ UTR of hepatotropic virus RNA and increase viral RNA stability leads to viral propagation [18, 20, 22]. Based on the above reports, we did *in silico* analysis of miRNAs targeting SARS and COVID-19 (recent isolates from different regions) to understand the pathophysiology and identify novel therapeutic targets.

**Table 1:** Details of SARS and COVID-19 isolates from different geographic locations, sequence length, and the number of human miRNA targets.

Virus type	GenBank ID	Location	Month and year of isolates/sequenced	Sequence Length (Nucleotides)	Number of miR Targets
SARS	AY338175.1	Taiwan	July 2003	29573	855
SARS	AY348314.1	Taiwan	July 2003	29573	855
SARS	AY291451.1	Taiwan	July 2003	29729	858
SARS	NC_004718.3	Canada	April 2003	29751	857
COVID -19	EPI_ISL_406798	Wuhan/China	December 2019	29866	893
COVID -19	EPI_ISL_403929	Wuhan/China	December 2019	29890	900
COVID -19	EPI_ISL_402121	Wuhan/China	December 2019	29891	898
COVID -19	EPI_ISL_402123	Wuhan/China	December 2019	29899	900
COVID -19	EPI_ISL_403931	Wuhan/China	December 2019	29889	903
COVID -19	EPI_ISL_403930	Wuhan/China	December 2019	29899	899
COVID -19	NC_045512.2	Wuhan (China)	January 2020	29903	900
COVID -19	MT007544.1	Australia	January 2020	29893	902
COVID -19	EPI_ISL_406862	Germany	January 2020	29782	896
COVID -19	EPI_ISL_403962	Thailand	January 2020	29848	897
COVID -19	EPI_ISL_412974	Italy	January 2020	29903	900
COVID -19	EPI_ISL_407893	Australia	January 2020	29782	898
COVID -19	EPI_ISL_406223	Arizona/USA	January 2020	29882	900
COVID -19	EPI_ISL_406597	France	January 2020	29809	901
COVID -19	EPI_ISL_420799	S. Korea	February 2020	29882	901
COVID -19	EPI_ISL_413214	Australia	February 2020	29782	899
COVID -19	EPI_ISL_419211	Isreal	February 2020	29851	897
COVID -19	MT050493.1	India	Fenruary 2020	29851	895
COVID -19	MT066176.1	Taiwan	February 2020	29870	900
COVID -19	EPI_ISL_418001	Portugal	March 2020	29763	895
COVID -19	EPI_ISL_417507	USA	March 2020	29782	898
COVID -19	MT159718.1	USA (Cruise A)	March 2020	29882	900
COVID -19	MT126808.1	Brazil	March 2020	29876	900
COVID -19	EPI_ISL_428847	Singapore	April 2020	29888	900
COVID -19	EPI_ISL_426565	Arizona/USA	April 2020	29882	897
COVID -19	EPI_ISL_420144	Georgia	April 2020	29833	900
COVID -19	EPI_ISL_427391	Turkey	April 2020	29895	899
COVID -19	EPI_ISL_429223	Switzerland	April 2020	29894	895
COVID -19	EPI_ISL_422601	Netherland	April 2020	29775	902

## MATERIALS AND METHODS

### Viral genome sequence retrieval, homology, and phylogenetic analyses

The complete genome sequences of the SARS and COVID-19 isolates were retrieved from the GenBank database. We retrieved four SARS and 29 COVID-19 sequences from NCBI and GISAID for for homology and phylogenetic analysis. Details of sequence identification are summarizing in Table 1. The sequences were aligned using the Multiple Sequence Alignment tool at Clustal Omega 1.2.3 on Geneious Prime 2020.1.1. The phylogenetic alignment tree generated using the neighbor-joining method.

### COVID-19 genome and human MiRNA target analysis

As previously mentioned above, miRNAs are known to target 3'UTR and coding sequences and prevent mRNA translation or degrade RNA. Keeping this in consideration, we used the whole viral genome sequence for miRNA target analysis. We used miRDB (http://www.mirdb.org/) software to identify novel human miRNAs targeting the COVID-19 viral genome [27, 28]. Furthermore, we used this data to correlate with the existing literature.

### GO, and KEGG pathway analysis

Gene Ontology (GO) and KEGG signaling pathway analyses were performed on human microRNAs targeting the COVID-19 genome using DIANA-miRPath v 3.0 (http://diana.imis.athena-innovation.gr/DianaTools/index.php) [29]. We used miRNAs with a target score above 90 because these miRNAs have high probability of being real targets [27, 28].

## RESULTS

### Viral genome sequence homology and phylogenetic analyses

The sequences of the COVID-19 isolates have been stored, compiled, and analyzed by various sources; one of the major resources for these sequences has been the Global Initiative on Sharing All Influenza Data (GISAID), which is a public-private initiative initially made for sharing sequences of influenza data. The organization provides a complete daily analysis of the viruses uploaded by the various researchers across the Globe. The COVID-19 belongs to a family of RNA viruses, which are very stable, unlikely other RNA viruses common to us, such as Human Immunodeficiency Virus (HIV), and Foot and Mouth Diseases Virus (FMDV).

**Table 2:** Sequence homology between the SARS and COVID-19 isolates from different geographic locations.

	AY291451.1	NC_004718.3	AY338175.1	AY348314.1	MT007544.1	EPI_ISL_429223	EPI_ISL_418001	EPI_ISL_420144	EPI_ISL_428847	EPI_ISL_427391	EPI_ISL_426565	EPI_ISL_403931	EPI_ISL_422601	MT050493.1	EPI_ISL_413214	EPI_ISL_419211	EPI_ISL_417507	EPI_ISL_406862	EPI_ISL_420799	EPI_ISL_402123	EPI_ISL_406223	EPI_ISL_407893	EPI_ISL_406597	EPI_ISL_406798	MT066176.1	MT126808.1	MT159718.1	EPI_ISL_402121	EPI_ISL_412974	EPI_ISL_403930	EPI_ISL_403962	EPI_ISL_403929	NC_045512.2
AY291451.1		100	100	100	78.8	78.8	78.7	78.7	78.8	78.7	78.8	78.8	78.8	78.8	78.8	78.8	78.8	78.8	78.8	78.8	78.8	78.8	78.8	78.8	78.8	78.8	78.8	78.8	78.8	78.8	78.8	78.8	78.8
NC_004718.3	100		100	100	78.8	78.8	78.7	78.7	78.8	78.7	78.8	78.8	78.7	78.8	78.8	78.8	78.7	78.8	78.8	78.8	78.8	78.8	78.7	78.8	78.8	78.8	78.8	78.8	78.8	78.8	78.8	78.8	78.8
AY338175.1	100	100		100	78.7	78.7	78.7	78.7	78.7	78.7	78.7	78.7	78.7	78.7	78.7	78.7	78.7	78.7	78.7	78.7	78.7	78.7	78.7	78.7	78.7	78.7	78.7	78.7	78.7	78.7	78.7	78.7	78.7
AY348314.1	100	100	100		78.7	78.7	78.7	78.7	78.7	78.7	78.7	78.7	78.7	78.7	78.7	78.7	78.7	78.7	78.7	78.7	78.7	78.7	78.7	78.7	78.7	78.7	78.7	78.7	78.7	78.7	78.7	78.7	78.7
MT007544.1	78.8	78.8	78.7	78.7		99.9	99.9	99.9	99.9	99.8	99.9	99.9	99.9	99.9	99.9	99.9	99.9	99.9	99.9	99.9	99.9	99.9	100	99.9	99.9	99.9	99.9	99.9	100	100	100	100	100
EPI_ISL_429223	78.8	78.8	78.7	78.7	99.9		100	100	99.9	99.9	100	99.9	100	99.9	100	100	100	100	100	100	100	100	100	100	100	100	100	100	100	100	100	100	100
EPI_ISL_418001	78.7	78.7	78.7	78.7	99.9	100		100	99.9	100	100	100	100	100	100	100	100	100	100	100	100	100	100	100	100	100	100	100	100	100	100	100	100
EPI_ISL_420144	78.7	78.7	78.7	78.7	99.9	100	100		99.9	99.9	100	100	100	100	100	100	100	100	100	100	100	100	100	100	100	100	100	100	100	100	100	100	100
EPI_ISL_428847	78.8	78.8	78.7	78.7	99.9	99.9	99.9	99.9		99.9	99.9	100	99.9	100	100	100	100	100	100	100	100	100	100	100	100	100	100	100	100	100	100	100	100
EPI_ISL_427391	78.7	78.7	78.7	78.7	99.8	99.9	100	99.9	99.9		99.9	99.9	100	99.9	99.9	99.9	99.9	100	99.9	99.9	99.9	100	99.9	99.9	99.9	99.9	99.9	99.9	99.9	99.9	99.9	99.9	99.9
EPI_ISL_426565	78.8	78.8	78.7	78.7	99.9	100	100	100	99.9	99.9		99.9	100	99.9	100	100	100	100	100	100	100	100	100	100	100	100	100	100	100	100	100	100	100
EPI_ISL_403931	78.8	78.8	78.7	78.7	99.9	99.9	100	100	100	99.9	99.9		100	100	100	100	100	100	100	100	100	100	100	100	100	100	100	100	100	100	100	100	100
EPI_ISL_422601	78.8	78.7	78.7	78.7	99.9	100	100	100	99.9	100	100	100		100	100	100	100	100	100	100	100	100	100	100	100	100	100	100	100	100	100	100	100
MT050493.1	78.8	78.8	78.7	78.7	99.9	99.9	100	100	100	99.9	99.9	100	100		100	100	100	100	100	100	100	100	100	100	100	100	100	100	100	100	100	100	100
EPI_ISL_413214	78.8	78.8	78.7	78.7	99.9	100	100	100	100	99.9	100	100	100	100		100	100	100	100	100	100	100	100	100	100	100	100	100	100	100	100	100	100
EPI_ISL_419211	78.8	78.8	78.7	78.7	99.9	100	100	100	100	99.9	100	100	100	100	100		100	100	100	100	100	100	100	100	100	100	100	100	100	100	100	100	100
EPI_ISL_417507	78.8	78.7	78.7	78.7	99.9	100	100	100	100	99.9	100	100	100	100	100	100		100	100	100	100	100	100	100	100	100	100	100	100	100	100	100	100
EPI_ISL_406862	78.8	78.8	78.7	78.7	99.9	100	100	100	100	100	100	100	100	100	100	100	100		100	100	100	100	100	100	100	100	100	100	100	100	100	100	100
EPI_ISL_420799	78.8	78.8	78.7	78.7	99.9	100	100	100	100	99.9	100	100	100	100	100	100	100	100		100	100	100	100	100	100	100	100	100	100	100	100	100	100
EPI_ISL_402123	78.8	78.8	78.7	78.7	99.9	100	100	100	100	99.9	100	100	100	100	100	100	100	100	100		100	100	100	100	100	100	100	100	100	100	100	100	100
EPI_ISL_406223	78.8	78.8	78.7	78.7	99.9	100	100	100	100	99.9	100	100	100	100	100	100	100	100	100	100		100	100	100	100	100	100	100	100	100	100	100	100
EPI_ISL_407893	78.8	78.8	78.7	78.7	99.9	100	100	100	100	100	100	100	100	100	100	100	100	100	100	100	100		100	100	100	100	100	100	100	100	100	100	100
EPI_ISL_406597	78.8	78.7	78.7	78.7	100	100	100	100	100	99.9	100	100	100	100	100	100	100	100	100	100	100	100		100	100	100	100	100	100	100	100	100	100
EPI_ISL_406798	78.8	78.8	78.7	78.7	99.9	100	100	100	100	99.9	100	100	100	100	100	100	100	100	100	100	100	100	100		100	100	100	100	100	100	100	100	100
MT066176.1	78.8	78.8	78.7	78.7	99.9	100	100	100	100	99.9	100	100	100	100	100	100	100	100	100	100	100	100	100	100		100	100	100	100	100	100	100	100
MT126808.1	78.8	78.8	78.7	78.7	99.9	100	100	100	100	99.9	100	100	100	100	100	100	100	100	100	100	100	100	100	100	100		100	100	100	100	100	100	100
MT159718.1	78.8	78.8	78.7	78.7	99.9	100	100	100	100	99.9	100	100	100	100	100	100	100	100	100	100	100	100	100	100	100	100		100	100	100	100	100	100
EPI_ISL_402121	78.8	78.8	78.7	78.7	99.9	100	100	100	100	99.9	100	100	100	100	100	100	100	100	100	100	100	100	100	100	100	100	100		100	100	100	100	100
EPI_ISL_412974	78.8	78.8	78.7	78.7	100	100	100	100	100	99.9	100	100	100	100	100	100	100	100	100	100	100	100	100	100	100	100	100	100		100	100	100	100
EPI_ISL_403930	78.8	78.8	78.7	78.7	100	100	100	100	100	99.9	100	100	100	100	100	100	100	100	100	100	100	100	100	100	100	100	100	100	100		100	100	100
EPI_ISL_403962	78.8	78.8	78.7	78.7	100	100	100	100	100	99.9	100	100	100	100	100	100	100	100	100	100	100	100	100	100	100	100	100	100	100	100		100	100
EPI_ISL_403929	78.8	78.8	78.7	78.7	100	100	100	100	100	99.9	100	100	100	100	100	100	100	100	100	100	100	100	100	100	100	100	100	100	100	100	100		100
NC_045512.2	78.8	78.8	78.7	78.7	100	100	100	100	100	99.9	100	100	100	100	100	100	100	100	100	100	100	100	100	100	100	100	100	100	100	100	100	100	

**Figure 1.**
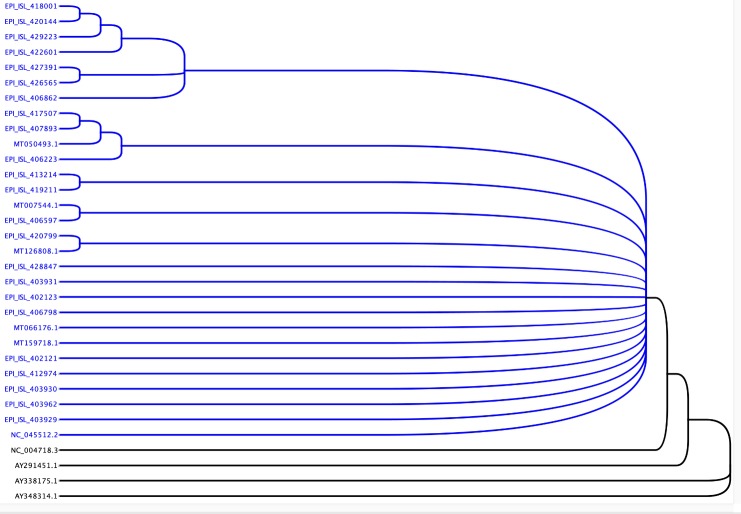
**Phylogenetic analysis of Coronavirus isolates from different geographic locations**. The phylogenetic analysis shows sequence relatedness among COVID-19 isolates (blue) and SARS isolates (black).

However, GISAID showed a constant drift in the COVID-19 population-based upon three specific mutations in its genome. To assimilate different sequences, we chose 29 genome sequences of COVID-19 from five continents covering 17 countries (Table 1). To gather the most diverse sequences, we collected these sequences based upon their date of isolation. Among 29 viral sequences, six isolated in 2019, and the remaining 23 were isolated at different months of 2020 from January to April (Table 1). These 29 COVID-19 sequences were compared with the four SARS genome sequences to evaluate the differences between the COVID-19 and SARS and among different isolates of COVID-19. The sequences were analyzed using Geneious Prime 2020.1.1. The data suggested the SARS genomes are approximately 78.7% similar to the COVID-19 sequences (Table 2). All 29 sequences of COVID-19 are very similar (99.9% - 100% sequence similarity). Phylogenetic analysis showed that COVID-19 is closely related to SARS isolates but still genetically different (Fig. 1). All 29 COVID-19 isolates are close to each other with little change in sequence. Since variability among different COVID-19 isolates was minimal, we calculated the number of different individual nucleotides in each COVID-19 isolates that revealed a total nucleotide difference among these COVID-19 is from 1-55 nucleotides in the entire genome of approximately 29kb (Supplementary Table 1). The most diverse sequence that has a difference of 55 nucleotides were between the isolates from Australia (MT007544.1) and Turkey (EPI_ISL_427391) isolated in 2019 and April 2020, respectively (Fig. 1 & Table 2, Supplementary Table 1).

**Table 3:** List of human miRNAs with higher target score (above 94), the number of binding sites, and miRNAs seed binding site on COVID-19 isolates.

miRNAs	Target Score	Number of Sites and Seed locations of miRNAs and COVID-19 genome binding sites
miR-15b-5p miR-15a-5p	99	16 SITES (3163, 5384, 8458, 8614, 13090, 14562, 14781, 19857, 24094, 24634, 25683, 26723, 28921, 28935, 28938, 29023) (Note: miR-15b-5p, and miR-15a-5p have same target site)
miR-548c-5p	97	15 SITES (2733, 4025, 4531, 6783, 7774, 9508, 10962, 11641, 11672, 12950, 13644, 20196, 21886, 23026, 25807)
miR-548d-3p	94	13 SITES (6960, 7245, 7272, 8927, 11540, 13459, 15517, 15814, 18367, 21100, 22217, 22583, 26653)
miR-409-3p	96	12 SITES (4990, 8386, 11785, 12403, 12525, 17285, 19760, 19803, 20759, 20829, 28767, 29694)
miR-30b-5p	95	14 SITES (3451, 4974, 7939, 9354, 10426, 11657, 16863, 19567, 19710, 20069, 20360, 26729, 27955, 28140)
miR-505-3p	95	11 SITES (152, 8488, 10609, 10792, 14208, 15648, 17580, 18123, 18156, 18612, 18906)

### MicroRNAs targeting SARS and COVID-19 genome 

**Figure 2.**
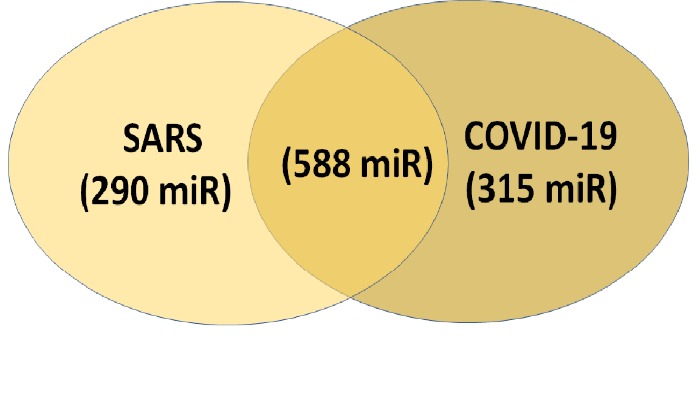
Common and different human miRNAs targeting SARS and COVID-19 isolates from different geographic locations.

As we mentioned above, we performed miRNA analysis on the whole genome of SARS and COVID-19 due to the efficiency of miRNAs targeting both 3'UTR and coding region. Our analysis found some interesting results. We found 848 common miRNAs targeting the SARS genome (four isolates, NC_004718.3, AY291451, AY338175, AY348314) and 873 common microRNAs targeting the 29 isolates of COVID-19 genome (Supplementary Table 2). Out of a total of 848 miRNAs from SARS, only 558 commonly present in all COVID-19 isolates (Fig. 2, Supplementary Table 2). Interestingly, 315 miRNAs are unique for COVID-19 isolates and 290 miRNAs unique to SRAS (Fig. 2). Furthermore, the COVID-19 targeting miRNAs with a higher target score (above 94) showed more than ten target sites and maximum complementary miRNA-RNA seed paring (~6-8 nucleotide seed base pairing) (Table 3). We also noted that out of 29 COVID-19 isolates, 19 have identical miRNA targets (Table 4). Ten isolates have unique miRNAs targets. Among ten isolates, Netherland (EPI_ISL_422601), Australia (EPI_ISL_413214), and Wuhan (EPI_ISL_403931) showed six, four, and four unique miRNAs targets respectively. The detail is given in the table (Table 4).

**Table 4:** Summary of important findings on human miRNAs targeting SARS and COVID-19 genome.

Serial. No	Important findings on human miRNAs targeting Coronavirus
1	848 miRNAs are common in SARS
2	873 miRNAs are common inCOVID-19
3	558 miRNAs are common between SARS and COVID-19
4	315 miRNAs are unique to COVID-19
5	290 miRNAs are unique to SARS
6	10 COVID-19 isolates have some unique miR targets
7	MT050493.1 (India): 1 unique miRNA (hsa-miR-449c-3p)
8	MT007544.1 (Australia): 2 unique miRNAs (hsa-miR-4538, hsa-miR-4453)
9	EPI_ISL_402121 (Wuhan/China): 1 unique miRNA (hsa-miR-5590-5p)
10	EPI_ISL_402123 (Wuhan/China): 1 unique miRNA (hsa-miR-106a-3p)
11	EPI_ISL_420799 (South Korea): 1 unique miRNA (hsa-miR-4641)
12	EPI_ISL_427391 (Turkey): 1 unique miRNA (hsa-miR-496)
13	EPI_ISL_429223 (Switzerland): 1 unique miRNA (hsa-miR-146b-3p)
14	EPI_ISL_403931 (Wuhan): 4 unique miRNAs (hsa-miR-4474-3p, hsa-miR-6762-3p, hsa-miR-10401-5p, hsa-miR-4304)
15	EPI_ISL_413214 (Australia): 4 unique miRNAs (hsa-miR-5088-5p, hsa-miR-9900, hsa-miR-3677-5p, hsa-miR-892c-5p)
16	EPI_ISL_422601 (Netherland): 6 unique miRNAs (hsa-miR-4666a-3p, hsa-miR-98-3p, hsa-let-7b-3p, hsa-let-7a-3p, hsa-miR-381-3p, hsa-miR-300)

**Table 5:** Human miRNAs targeting the COVID-19 genome regulating KEGG pathway.

KEGG pathway	p-value	#genes	#miRNAs
Proteoglycans in cancer	5.75E-08	145	76
Hippo signaling pathway	1.04E-07	113	74
Arrhythmogenic right ventricular cardiomyopathy (ARVC)	6.54E-07	57	72
Adherens junction	6.54E-07	62	75
Renal cell carcinoma	2.40E-06	56	74
Wnt signaling pathway	2.99E-06	107	76
Fatty acid biosynthesis	1.25E-05	9	50
ECM-receptor interaction	1.25E-05	56	70
Axon guidance	1.58E-05	94	75
FoxO signaling pathway	4.68E-05	100	76
Ubiquitin mediated proteolysis	5.75E-05	102	76
Pathways in cancer	6.76E-05	275	76
ErbB signaling pathway	8.02E-05	66	75
Pancreatic cancer	0.000165	53	73
TGF-beta signaling pathway	0.000234	57	73
Focal adhesion	0.000234	147	74
Rap1 signaling pathway	0.000234	148	76
Gap junction	0.000753	64	76
Long-term depression	0.000962	45	73
N-Glycan biosynthesis	0.001119	33	69
Prion diseases	0.001166	20	66
Endocytosis	0.001469	140	75
Fatty acid metabolism	0.001547	31	69
Endometrial cancer	0.001567	41	72
Signaling pathways regulating pluripotency of stem cells	0.001567	99	76
Prostate cancer	0.001769	66	75
Colorectal cancer	0.002458	49	72
Cell cycle	0.002703	89	73
PI3K-Akt signaling pathway	0.002703	225	76
Melanoma	0.00405	54	73
Circadian rhythm	0.00591	26	70
Prolactin signaling pathway	0.006364	50	75
Adrenergic signaling in cardiomyocytes	0.006716	97	77
Glycosaminoglycan biosynthesis - heparan sulfate / heparin	0.006964	17	62
Dorso-ventral axis formation	0.011682	23	73
AMPK signaling pathway	0.012171	87	75
Glioma	0.012308	45	72
Tight junction	0.012616	98	76
Thyroid hormone signaling pathway	0.01495	79	72
Morphine addiction	0.01495	63	73
Oocyte meiosis	0.01495	79	75
Ras signaling pathway	0.01495	145	76
Lysine degradation	0.016507	33	66
Amphetamine addiction	0.016687	45	72
Sphingolipid signaling pathway	0.016687	79	76
Glutamatergic synapse	0.016687	77	76
mRNA surveillance pathway	0.01713	64	74
RNA transport	0.01833	112	75
MAPK signaling pathway	0.018745	166	77
Chronic myeloid leukemia	0.01925	51	74
Estrogen signaling pathway	0.022066	65	76
GABAergic synapse	0.023522	59	73
p53 signaling pathway	0.026352	48	73
Biosynthesis of unsaturated fatty acids	0.027342	15	49
mTOR signaling pathway	0.031797	45	70
Regulation of actin cytoskeleton	0.037298	139	75
Protein processing in endoplasmic reticulum	0.038084	112	74
cAMP signaling pathway	0.038084	130	76
Oxytocin signaling pathway	0.038084	104	77
Glycosaminoglycan biosynthesis - keratan sulfate	0.039424	12	23
Central carbon metabolism in cancer	0.04664	46	70
Melanogenesis	0.04852	68	76

### GO and KEGG pathway analysis of miRNAs targeting COVID-19

To identify the functional relevance of human miRNAs targeting COVID-19, we performed Kyoto Encyclopedia of Genes and Genomes (KEGG) pathway annotation and GO analysis. The KEGG annotation analysis data reveal that these miRNAs plays important in various Cancer signaling (e.g Renal, Pancreatic, Prostate, Colorectal, Melanoma ), Hippo signaling pathway, cardiomyopathy, Wnt signaling, Circadian rhythm, stem cell signaling, Adrenergic signaling in cardiomyocytes and number of other signaling pathways (Table 5). GO analysis data showed more than 72 biological processes were associated with the miRNAs targeting COVID-19 (Table 6). The biological processes such as organelle, ion binding, cellular, biosynthesis process, protein complex, immune response, and viral process are regulated by human miRNAs targeting the COVID-19 genome. Details of KEGG annotation and GO analysis are shown in Table 5, and Table 6, respectively.

## DISCUSSION

Host cellular miRNAs are known to play an antiviral role in the number of published studies [18-23]. In this study, we performed *in silico* analysis of human cellular miRNAs targeting SARS and COVID-19 (isolates) genome and identified some novel miRNAs. We identified number (558) of common human cellular miRNAs targeting both SARS and COVID-19 genome. Top 10 common miRNAs have a target score of ≥95 (target score between 99-95), and each miRNA contains more than at least ten sites in the targeting viral genome (Table 3), indicating possible antiviral property for coronavirus infection (for both SARS and COVID-19). It will be interesting to verify these miRNA *in-vitro* and *in-vivo* animal models to use as a therapeutic target in the future. Cocktail of multiple miRNAs mimics through the intranasal route will be useful in coronavirus infection. These human miRNAs targeting coronavirus can be useful to combat any future outbreaks. In a previous report, host cellular miRNAs-181 binds to the ORF-4 region at the viral genome of porcine reproductive and respiratory syndrome virus (PRRSV) to inhibit its replication [17]. One step further, Guo et al. (2013) delivered intranasal inhalation of miR-181 mimics to slow down the progression of PRRSV in an experimental porcine model [17]. Another study used intranasal inoculation of miR-130 mimic to protect piglets from lethal challenge of PRRSV [30]. Similarly, the intranasal administration of chemically modified five miRNA mimics protected mice from H1N1 viral infection [31].

**Table 6:** Human miRNAs targeting the COVID-19 genome regulating GO pathway.

GO Category	p-value	#genes	#miRNAs
organelle	1.26E-49	980	64
ion binding	5.53E-28	611	64
cellular nitrogen compound metabolic process	1.82E-23	474	63
biosynthetic process	1.36E-13	388	42
neurotrophin TRK receptor signaling pathway	7.06E-13	44	44
protein binding transcription factor activity	1.83E-12	75	29
Fc-epsilon receptor signaling pathway	5.76E-12	32	24
protein complex	6.82E-11	385	64
gene expression	4.82E-10	70	35
cellular protein modification process	7.10E-10	232	41
molecular_function	7.10E-10	1552	66
extracellular matrix disassembly	1.72E-09	26	14
viral process	1.82E-09	60	49
symbiosis, encompassing mutualism through parasitism	1.82E-09	66	49
small molecule metabolic process	4.04E-09	229	57
catabolic process	1.70E-08	197	58
collagen catabolic process	3.52E-08	22	12
cellular component assembly	5.85E-08	141	36
cellular_component	7.90E-08	1559	66
macromolecular complex assembly	1.77E-07	101	36
blood coagulation	8.36E-07	55	26
nucleic acid binding transcription factor activity	1.97E-06	107	38
cytosol	3.58E-06	267	57
protein complex assembly	4.08E-06	88	48
epidermal growth factor receptor signaling pathway	1.64E-05	31	23
enzyme binding	1.92E-05	130	51
extracellular matrix organization	2.18E-05	51	21
nucleoplasm	2.49E-05	122	56
cellular protein metabolic process	3.33E-05	49	29
xenobiotic metabolic process	4.07E-05	23	21
immune system process	4.82E-05	160	36
nucleobase-containing compound catabolic process	6.69E-05	92	53
endoplasmic reticulum lumen	0.000154305	29	16
response to stress	0.000159934	210	39
innate immune response	0.000224462	80	28
microtubule organizing center	0.000453748	56	43
Fc-gamma receptor signaling pathway involved in phagocytosis	0.00093232	12	17
toll-like receptor TLR1:TLR2 signaling pathway	0.001615504	11	15
toll-like receptor TLR6:TLR2 signaling pathway	0.001615504	11	15
fibroblast growth factor receptor signaling pathway	0.001615504	26	23
mitotic cell cycle	0.001685088	39	45
glutathione derivative biosynthetic process	0.001906053	7	12
DNA metabolic process	0.00191818	79	34
biological_process	0.00191818	1484	66
phosphatidylinositol-mediated signaling	0.002737027	20	22
toll-like receptor 2 signaling pathway	0.005968204	12	17
cytoskeleton-dependent intracellular transport	0.007263134	17	15
toll-like receptor 4 signaling pathway	0.007263134	14	17
membrane organization	0.007346668	56	45
cellular response to jasmonic acid stimulus	0.007563711	3	1
cell motility	0.008091976	60	31
G2/M transition of mitotic cell cycle	0.010059905	20	36
cell-cell signaling	0.010959215	65	31
platelet degranulation	0.011941199	11	15
protein N-linked glycosylation via asparagine	0.012823473	14	14
homeostatic process	0.013223078	81	27
post-translational protein modification	0.013916886	18	20
toll-like receptor 10 signaling pathway	0.015857167	9	14
cell death	0.015857167	85	25
substrate-dependent cell migration, cell extension	0.018717628	5	9
nervous system development	0.019812789	51	26
toll-like receptor 9 signaling pathway	0.021790835	10	16
RNA binding	0.021790835	168	42
platelet activation	0.023223454	22	20
extracellular matrix structural constituent	0.034117778	16	4
transcription coactivator activity	0.034117778	41	23
cytoskeletal protein binding	0.036370846	71	34
toll-like receptor 5 signaling pathway	0.039177086	9	14
axon guidance	0.040371436	49	21
cAMP metabolic process	0.043778349	3	9
TRIF-dependent toll-like receptor signaling pathway	0.045227284	9	14

**Table 7:** List of selected human miRNAs targeting the COVID-19 genome down-regulated with age and underlying conditions.

miRNA	Decrease Expression in age related diseases (Human)	Reference
miR-15b-5p	Coronary Artery Disease	Zhu et al 2017 [37]
miR-15a-5p	Kidney disease	Shang et al 2019 [38]
miR-548c-5p	Colorectal Cancer	Peng et al 2018 [49]
miR-548d-3p	Osteosarcoma	Chen et al 2019 [50]
miR-409-3p	Osteosarcoma	Wu et al 2019 [51]
miR-30b-5p	Plasma (Aging)	Hatse et al 2014 [52]
miR-505-3p	Prostate cancer	Tang et al 2019 [53]
miR-520c-3p	Obesity/diabetes	Ortega et al 2013 [39]
miR-30e-3p	Myocardial Injury	Wang et al 2017 [40]
miR-23c	Hepatocellular carcinoma	Zhang et al 2018 [41]
miR-30d-5p	Non-small cell lung cancer	Gao et al, 2018 [42]
miR-4684-3p	Colorectal cancer	Wu et al, 2015 [43]
miR-518a-5p	Gastrointestinal tumors	Shi et al, 2016 [44]

In humans, coronavirus infection is pervasive and usually causes common cold-like symptoms, but some of them can lead to serious illness. Previous coronavirus outbreak in 2002, known as SARS-CoV spread worldwide but was contained quickly [9]. On the other hand, the current coronavirus (COVID-19) is highly contagious and spread worldwide rapidly and became a global pandemic [8,11]. It will be interesting to know what separates these two viruses based on the genomic sequence. Ours (Table 2 and Fig. 1) and other data [32-34] of SARS-CoV and COVID-19 genome sequence analysis showed sequence homology of around ~78.7%, which was obvious, but our miRNA target scan data showed striking results. We found considerable changes in the number of human miRNAs targeting SARS-CoV (848 miRNAs) and COVID-19 (~873 miRNAs). Moreover, we also found unique miRNAs for SARS-CoV (290 miRNAs) and COVID-19 (315 miRNAs) isolates. At nucleotide (Table 2) and phylogenetic levels (Fig. 1), both viruses look closely related, but host cellular miRNAs target comparison differences are much more significant. This might be one of the reasons for COVID-19 to increase infectivity and easy viral propagation compared to SARS-CoV. The COVID-19 recent isolates (twenty-nine different isolates) are closely related at nucleotide, phylogenetic (Fig. 1 and Table 2), and host cellular miRNAs target level (Fig. 2 and Supplementary Table 2). The Netherland (EPI_ISL_422601), Australia (EPI_ISL_413214), and Wuhan (EPI_ISL_403931) isolates have more mutation and few different host cellular miRNAs targeting sites compared to other 26 COVID-19 isolates (Table 4). In our study, we used 29 isolates from different geographical reagion, which informs that COVID-19 nucleotide sequences and thus the human miRNAs target sites are not changing among the isolates/viruses (Table 4). In most of the COVID-19 cases, the virus entered a country from different geographical locations. At the time of writing this paper, only the USA and China had sequenced multiple viruses isolates from a different region. There is a need to sequence more of the COVID-19 viral genome from different parts of each country before coming to any conclusion.

The most important and striking feature of COVID-19 is the increased case fatality rate in aged individuals. The CDC reports 45% of cases requiring hospitalization are patients ≥65 years old [12]. Furthermore, 80% of COVID-19 related deaths occur in these patients (≥65 years old) [12]. Moreover, individuals with underlying conditions such as cardiovascular, chronic lung disease, diabetes, kidney, liver diseases, and cancer are at higher risk for severe illness and mortality. On the contrary, <1% mortality is observed in patients between 20-54 years of age or younger [12]. Based on miRNA target analysis and published literature, we hypothesized that the COVID-19 genome replicates and propagates viral particles easily in the aged individuals compared to younger due to the overall low abundance of miRNA expression with age. Recent human studies reported that the overall expression of miRNAs levels goes down with age [16, 35, 36]. For example, Huan et al. (2018) reported that 81% (103 miRNAs) microRNAs were negatively, and 19% (24 miRNAs) positively correlated with age in human peripheral blood [36]. We speculate that in younger individuals with COVID-19 infection, host cellular miRNAs bind to the complementary site of the viral RNA genome and prevent its replication; however, in an aged individual, this mechanism is not that efficient leads to accumulation of viral particle and severe illness. This might be true for underlying conditions as well. Some of the miRNAs targeting the COVID-19 genome showed low abundance (down-regulated) with some underlying conditions (Table 7). For example, miR-15b-5p (Coronary artery disease) [37], miR-15a-5p (Kidney disease) [38], miR-520c-3p (obesity/diabetes) [39], miR-30e-3p (Myocardial Injury) [40], miR-23c (hepatocellular carcinoma) [41], miR-30d-5p (non-small cell lung cancer) [42], miR-4684-3p (colorectal cancer) 43], and miR-518a-5p (Gastrointestinal stromal tumors) [44] down-regulated in pathophysiological condition.

To identify the biological relevance of COVID-19 targeting human cellular miRNAs, KEGG pathway annotation, and GO analysis was performed on target gene pools. KEGG annotation data showed that COVID-19 targeting human miRNAs plays an important role in various cancer signaling, Hippo signaling pathways, cardiac (e.g cardiomyopathy, Adrenergic signaling in cardiomyocytes), cell cycle, FoxO and number of other signaling pathways (Table.5). Furthermore, GO pathway annotation analysis showed that the COVID-19 targeting human miRNAs involved in important signaling dysregulated during age-related complications, such as biosynthesis, metabolic, cellular protein modification, and cellular component assembly. Most importantly, we also found that signaling related to immune response, Fc-epsilon receptor signaling pathway, viral process, and symbiosis, encompassing mutualism through parasitism signaling affected by the COVID-19 targeting human cellular miRNAs (Table.6). The signaling mentioned above is important to fight against viral infections [45-48]. Both KEGG and GO pathway analysis revealed that COVID-19 targeting human cellular miRNAs are involved in the number of age-related complications. Impaired GO and KEGG pathways may be due to low abundance miRNA and might be one of the contributing factors for the increasing severity and mortality of the COVID-19 infection in aged individuals and with other underlying conditions.

The dual role of host cellular miRNAs cannot be denied in the viral replication or inhibition. Host miRNAs can have both functions, antiviral, which is beneficial to host, or miRNA-viral genome interaction can increase stability and beneficiary for viral propagation [18, 20, 22]. In a viral infection, we hypothesized that host cellular miRNAs predominantly have antiviral activity. Though possible, positive regulation of viral genome translation by miRNAs is less likely. Our data and hypothesis are based on *in silico* analysis and realize that not all miRNAs targets will be real. Considering the many miRNAs targets identified in our *in-silico* analysis, even if 5% of total miRNAs targets are real (based on target score), it is possible that these could be used for therapeutic purposes. Further, *in vitro* and *in vivo* studies will be needed to validate some of these targets. However, in view of the current lack of effective treatment strategies, innovative approaches need to be considered.

## Supplementary Materials

The Supplemenantry data can be found online at: www.aginganddisease.org/EN/10.14336/AD.2020.0428.
