# Supplementary material for: COVID-19 Virulence in Aged Patients Might Be Impacted by the Host Cellular MicroRNAs Abundance/Profile
Source: Aging Dis. 2020 Apr 28;11(3):509–22. doi: 10.14336/AD.2020.0428 (PMC7220294; doi:10.14336/AD.2020.0428)
Supplement: Supplementary file 1 — The Supplemenantry data can be found online at: www.aginganddisease.org/EN/10.14336/AD.2020.0428. [file AD-11-3-509-s.pdf]

SUPPLEMENTARY DATA

COVID-19 Virulence in Aged Patients Might Be Impacted by the Host Cellular MicroRNAs Abundance/Profile

Sadanand Fulzele[1,2*], Bikash Sahay[3], Ibrahim Yusufu[1], Tae Jin Lee[4], Ashok Sharma[4], Ravindra Kolhe[5], Carlos M. Isales[1,2]

[1]Department of Medicine, Augusta University, Augusta, GA, USA. [2]Center for Healthy Aging, Augusta University, Augusta, GA, USA. [3]Department of Infectious Diseases and Immunology, University of Florida, Gainesville, FL, USA. [4]Center for Biotechnology and Genomic Medicine, Augusta University, Augusta, GA 30912, USA. [5]Departments of Pathology, Augusta University, Augusta, GA 30912, USA

© 2020. Fulzele S et al. Published online at http://www.aginganddisease.org/EN/10.14336/AD.2020.0428

SUPPLEMENTARY DATA

Supplementary Table 1. Number of nucleotide differences between the SARS and COVID-19 isolates.

	AY291451.1	NC_004718.3	AY338175.1	AY348314.1	MT007544.1	EPI_ISL_429223	EPI_ISL_418001	EPI_ISL_420144	EPI_ISL_428847	EPI_ISL_427391	EPI_ISL_426565	EPI_ISL_403931	EPI_ISL_422601	MT050493.1	EPI_ISL_413214	EPI_ISL_419211	EPI_ISL_417507	EPI_ISL_406862	EPI_ISL_420799	EPI_ISL_402123	EPI_ISL_406223	EPI_ISL_407893	EPI_ISL_406597	EPI_ISL_406798	MT066176.1	MT126808.1	MT159718.1	EPI_ISL_402121	EPI_ISL_412974	EPI_ISL_403930	EPI_ISL_403962	EPI_ISL_403929	NC_045512.2
**AY291451.1**		3	10	8	6372	6364	6358	6365	6362	6387	6368	6363	6352	6361	6351	6358	6352	6351	6362	6364	6360	6351	6358	6356	6361	6361	6363	6361	6361	6362	6358	6361	6361
**NC_004718.**	3		11	9	6374	6366	6360	6367	6364	6389	6370	6365	6354	6363	6353	6360	6354	6353	6364	6366	6362	6353	6360	6358	6363	6363	6365	6363	6363	6364	6360	6363	6363
**AY338175.1**	10	11		4	6365	6360	6360	6359	6356	6357	6361	6356	6356	6359	6355	6353	6356	6355	6355	6357	6353	6355	6356	6355	6354	6354	6356	6354	6354	6355	6354	6354	6354
**AY348314.1**	8	9	4		6365	6360	6360	6359	6356	6357	6361	6356	6356	6359	6355	6353	6356	6355	6355	6357	6353	6355	6356	6355	6354	6354	6356	6354	6354	6355	6354	6354	6354
**MT007544.**	6372	6374	6365	6365		22	21	20	21	55	22	19	20	19	18	17	18	16	16	16	17	15	13	15	15	15	15	15	13	14	13	13	13
**EPI_ISL_4292**	6364	6366	6360	6360	22		3	2	17	34	10	15	8	15	14	13	14	6	14	12	13	11	11	11	11	13	11	11	11	10	9	9	9
**EPI_ISL_4180**	6358	6360	6360	6360	21	3		1	16	10	9	14	7	14	13	9	13	5	13	11	12	10	10	10	10	12	10	10	10	9	8	8	8
**EPI_ISL_4201**	6365	6367	6359	6359	20	2	1		15	38	8	13	6	13	12	10	12	4	12	10	11	9	9	9	9	11	9	9	9	8	7	7	7
**EPI_ISL_4288**	6362	6364	6356	6356	21	17	16	15		47	17	14	15	14	11	10	13	11	9	11	10	10	10	10	10	10	10	10	8	9	8	8	8
**EPI_ISL_4273**	6387	6389	6357	6357	55	34	10	38	47		41	48	9	28	15	24	15	7	47	45	46	12	25	19	44	46	44	44	44	43	31	42	42
**EPI_ISL_4265**	6368	6370	6361	6361	22	10	9	8	17	41		15	8	15	14	13	14	6	14	12	13	11	11	11	11	13	11	11	11	10	9	9	9
**EPI_ISL_4039**	6363	6365	6356	6356	19	15	14	13	14	48	15		13	12	11	10	11	9	11	9	10	8	8	8	8	10	8	8	8	7	6	6	6
**EPI_ISL_4226**	6352	6354	6356	6356	20	8	7	6	15	9	8	13		13	12	8	12	4	12	10	11	9	9	9	9	11	9	9	9	8	7	7	7
**MT050493.**	6361	6363	6359	6359	19	15	14	13	14	28	15	12	13		11	10	7	9	11	9	6	4	8	8	8	10	8	8	8	7	6	6	6
**EPI_ISL_4132**	6351	6353	6355	6355	18	14	13	12	11	15	14	11	12	11		4	10	8	8	8	7	7	7	7	7	7	7	7	5	6	5	5	5
**EPI_ISL_4192**	6358	6360	6353	6353	17	13	9	10	10	24	13	10	8	10	4		6	4	7	7	6	3	5	4	5	6	6	6	4	5	3	4	4
**EPI_ISL_4175**	6352	6354	6356	6356	18	14	13	12	13	15	14	11	12	7	10	6		8	10	8	5	3	7	7	7	9	7	7	7	6	5	5	5
**EPI_ISL_4068**	6351	6353	6355	6355	16	6	5	4	11	7	6	9	4	9	8	4	8		8	6	7	5	5	5	5	7	5	5	5	4	3	3	3
**EPI_ISL_4207**	6362	6364	6355	6355	16	14	13	12	9	47	14	11	12	11	8	7	10	8		8	7	7	5	7	7	3	7	7	3	6	5	5	5
**EPI_ISL_4021**	6364	6366	6357	6357	16	12	11	10	11	45	12	9	10	9	8	7	8	6	8		7	5	5	5	5	7	5	5	5	4	3	3	3
**EPI_ISL_4062**	6360	6362	6353	6353	17	13	12	11	10	46	13	10	11	6	7	6	5	7	7	7		2	6	6	6	6	6	6	4	5	4	4	4
**EPI_ISL_4078**	6351	6353	6355	6355	15	11	10	9	10	12	11	8	9	4	7	3	3	5	7	5	2		4	4	4	6	4	4	4	3	2	2	2
**EPI_ISL_4065**	6358	6360	6356	6356	13	11	10	9	10	25	11	8	9	8	7	5	7	5	5	5	6	4		4	4	4	4	4	2	3	2	2	2
**EPI_ISL_4067**	6356	6358	6355	6355	15	11	10	9	10	19	11	8	9	8	7	4	7	5	7	5	6	4	4		4	6	4	4	4	3	2	2	2
**MT066176.**	6361	6363	6354	6354	15	11	10	9	10	44	11	8	9	8	7	5	7	5	7	5	6	4	4	4		6	4	4	4	3	2	2	2
**MT126808.**	6361	6363	6354	6354	15	13	12	11	10	46	13	10	11	10	7	6	9	7	3	7	6	6	4	6	6		6	6	2	5	4	4	4
**MT159718.**	6363	6365	6356	6356	15	11	10	9	10	44	11	8	9	8	7	6	7	5	7	5	6	4	4	4	4	6		4	4	3	2	2	2
**EPI_ISL_4021**	6361	6363	6354	6354	15	11	10	9	10	44	11	8	9	8	7	6	7	5	7	5	6	4	4	4	4	6	4		4	3	2	2	2
**EPI_ISL_4129**	6361	6363	6354	6354	13	11	10	9	8	44	11	8	9	8	5	4	7	5	3	5	4	4	2	4	4	2	4	4		3	2	2	2
**EPI_ISL_4039**	6362	6364	6355	6355	14	10	9	8	9	43	10	7	8	7	6	5	6	4	6	4	5	3	3	3	3	5	3	3	3		1	1	1
**EPI_ISL_4039**	6358	6360	6354	6354	13	9	8	7	8	31	9	6	7	6	5	3	5	3	5	3	4	2	2	2	2	4	2	2	2	1		0	0
**EPI_ISL_4039**	6361	6363	6354	6354	13	9	8	7	8	42	9	6	7	6	5	4	5	3	5	3	4	2	2	2	2	4	2	2	2	1	0		0
**NC_045512.**	6361	6363	6354	6354	13	9	8	7	8	42	9	6	7	6	5	4	5	3	5	3	4	2	2	2	2	4	2	2	2	1	0	0	

© 2020. Fulzele S et al. Published online at http://www.aginganddisease.org/EN/10.14336/AD.2020.0428

SUPPLEMENTARY DATA

Supplementary Table 2. List of common and unique human cellular miRNAs targeting SARS and COVID-19 isolates.

**Common miRNAs**	**Unique COVID-19 miRNAs Compared to SARS**	**Unique SARS miRNAs Compared to COVID-19**	**Common miRNAs in all COVID-19 isolates**
hsa-miR-4288	hsa-miR-4684-3p	hsa-miR-4672	hsa-miR-4288
hsa-miR-195-5p	hsa-miR-4490	hsa-miR-4293	hsa-miR-195-5p
hsa-miR-16-5p	hsa-miR-527	hsa-miR-4276	hsa-miR-16-5p
hsa-miR-15b-5p	hsa-miR-518a-5p	hsa-miR-466	hsa-miR-15b-5p
hsa-miR-15a-5p	hsa-miR-628-3p	hsa-miR-4715-5p	hsa-miR-15a-5p
hsa-miR-6838-5p	hsa-miR-141-3p	hsa-miR-7852-3p	hsa-miR-6838-5p
hsa-miR-497-5p	hsa-miR-200a-3p	hsa-miR-599	hsa-miR-497-5p
hsa-miR-424-5p	hsa-miR-6509-5p	hsa-miR-4516	hsa-miR-424-5p
hsa-miR-3133	hsa-miR-3671	hsa-miR-6895-3p	hsa-miR-3133
hsa-miR-21-3p	hsa-miR-4504	hsa-miR-5585-5p	hsa-miR-21-3p
hsa-miR-122b-3p	hsa-miR-582-3p	hsa-miR-5579-3p	hsa-miR-122b-3p
hsa-miR-559	hsa-miR-6124	hsa-miR-205-3p	hsa-miR-559
hsa-miR-196a-1-3p	hsa-miR-570-5p	hsa-miR-5701	hsa-miR-196a-1-3p
hsa-miR-548y	hsa-miR-548ba	hsa-miR-3137	hsa-miR-548y
hsa-miR-548w	hsa-miR-548ai	hsa-miR-493-3p	hsa-miR-548w
hsa-miR-548o-5p	hsa-miR-548ag	hsa-miR-302f	hsa-miR-548o-5p
hsa-miR-548h-5p	hsa-miR-4527	hsa-miR-6833-3p	hsa-miR-548h-5p
hsa-miR-548d-5p	hsa-miR-5684	hsa-miR-1248	hsa-miR-548d-5p
hsa-miR-548c-5p	hsa-miR-548n	hsa-miR-545-5p	hsa-miR-548c-5p
hsa-miR-548bb-5p	hsa-miR-6503-5p	hsa-miR-490-3p	hsa-miR-548bb-5p
hsa-miR-548b-5p	hsa-miR-4744	hsa-miR-877-3p	hsa-miR-548b-5p
hsa-miR-548ay-5p	hsa-miR-142-5p	hsa-miR-539-5p	hsa-miR-548ay-5p
hsa-miR-548au-5p	hsa-miR-6800-5p	hsa-miR-6806-3p	hsa-miR-548au-5p
hsa-miR-548ar-5p	hsa-miR-577	hsa-miR-3928-5p	hsa-miR-548ar-5p
hsa-miR-548am-5p	hsa-miR-605-5p	hsa-miR-224-5p	hsa-miR-548am-5p
hsa-miR-548ak	hsa-miR-5590-3p	hsa-miR-129-2-3p	hsa-miR-548ak
hsa-miR-548ae-5p	hsa-miR-552-5p	hsa-miR-129-1-3p	hsa-miR-548ae-5p
hsa-miR-548ad-5p	hsa-miR-6805-3p	hsa-miR-4481	hsa-miR-548ad-5p
hsa-miR-548aq-5p	hsa-miR-5691	hsa-miR-5590-5p	hsa-miR-548aq-5p
hsa-miR-302c-5p	hsa-miR-485-3p	hsa-miR-1537-5p	hsa-miR-302c-5p
hsa-miR-548a-5p	hsa-miR-539-3p	hsa-miR-3682-5p	hsa-miR-548a-5p
hsa-miR-548as-5p	hsa-miR-8063	hsa-miR-216b-5p	hsa-miR-548as-5p
hsa-miR-548ab	hsa-miR-302d-3p	hsa-miR-6822-3p	hsa-miR-548ab
hsa-miR-409-3p	hsa-miR-302c-3p	hsa-miR-1236-5p	hsa-miR-4684-3p
hsa-miR-548j-5p	hsa-miR-302b-3p	hsa-miR-5190	hsa-miR-409-3p
hsa-miR-548i	hsa-miR-302a-3p	hsa-miR-2115-3p	hsa-miR-548j-5p

© 2020. Fulzele S et al. Published online at http://www.aginganddisease.org/EN/10.14336/AD.2020.0428

SUPPLEMENTARY DATA

hsa-miR-548ap-5p	hsa-miR-325	hsa-miR-6072	hsa-miR-548i
hsa-miR-186-5p	hsa-miR-4483	hsa-miR-4326	hsa-miR-548ap-5p
hsa-miR-545-3p	hsa-miR-20a-3p	hsa-miR-183-5p	hsa-miR-186-5p
hsa-miR-6835-3p	hsa-miR-647	hsa-miR-4768-5p	hsa-miR-545-3p
hsa-miR-30c-5p	hsa-miR-5087	hsa-miR-4424	hsa-miR-6835-3p
hsa-miR-30b-5p	hsa-miR-6863	hsa-miR-448	hsa-miR-30c-5p
hsa-miR-6830-3p	hsa-miR-23c	hsa-miR-6881-3p	hsa-miR-30b-5p
hsa-miR-30e-5p	hsa-miR-23b-3p	hsa-miR-4268	hsa-miR-6830-3p
hsa-miR-30d-5p	hsa-miR-23a-3p	hsa-miR-7107-3p	hsa-miR-30e-5p
hsa-miR-30a-5p	hsa-miR-30e-3p	hsa-miR-6753-3p	hsa-miR-30d-5p
hsa-miR-3686	hsa-miR-30d-3p	hsa-miR-501-5p	hsa-miR-30a-5p
hsa-miR-505-3p	hsa-miR-30a-3p	hsa-miR-3119	hsa-miR-3686
hsa-miR-632	hsa-miR-122b-5p	hsa-miR-4323	hsa-miR-505-3p
hsa-miR-548z	hsa-miR-6719-3p	hsa-miR-6829-3p	hsa-miR-632
hsa-miR-548h-3p	hsa-miR-4733-3p	hsa-miR-320e	hsa-miR-548z
hsa-miR-548d-3p	hsa-miR-4714-5p	hsa-miR-6891-3p	hsa-miR-548h-3p
hsa-miR-548bb-3p	hsa-miR-145-3p	hsa-miR-7853-5p	hsa-miR-548d-3p
hsa-miR-548ac	hsa-miR-6875-5p	hsa-miR-105-5p	hsa-miR-548bb-3p
hsa-miR-548m	hsa-miR-520d-3p	hsa-miR-8069	hsa-miR-548ac
hsa-miR-374a-5p	hsa-miR-520a-3p	hsa-miR-6773-3p	hsa-miR-548m
hsa-miR-4778-3p	hsa-miR-1293	hsa-miR-580-5p	hsa-miR-374a-5p
hsa-miR-4422	hsa-miR-4719	hsa-miR-7848-3p	hsa-miR-4778-3p
hsa-miR-5688	hsa-miR-411-3p	hsa-miR-1251-5p	hsa-miR-4422
hsa-miR-548as-3p	hsa-miR-379-3p	hsa-miR-19b-3p	hsa-miR-4490
hsa-miR-7159-5p	hsa-miR-4774-5p	hsa-miR-19a-3p	hsa-miR-527
hsa-miR-6512-5p	hsa-miR-508-3p	hsa-miR-3194-5p	hsa-miR-518a-5p
hsa-miR-29c-3p	hsa-miR-3614-3p	hsa-miR-4732-5p	hsa-miR-5688
hsa-miR-29b-3p	hsa-miR-34c-3p	hsa-miR-4324	hsa-miR-548as-3p
hsa-miR-633	hsa-miR-6828-5p	hsa-miR-4317	hsa-miR-7159-5p
hsa-miR-1226-3p	hsa-miR-520e-3p	hsa-miR-4752	hsa-miR-6512-5p
hsa-miR-4310	hsa-miR-520c-3p	hsa-miR-512-5p	hsa-miR-29c-3p
hsa-miR-495-3p	hsa-miR-520b-3p	hsa-miR-2278	hsa-miR-29b-3p
hsa-miR-4775	hsa-miR-373-3p	hsa-miR-4279	hsa-miR-633
hsa-miR-374b-5p	hsa-miR-372-3p	hsa-miR-133b	hsa-miR-1226-3p
hsa-miR-1283	hsa-miR-4708-5p	hsa-miR-133a-3p	hsa-miR-4310
hsa-miR-4642	hsa-miR-3606-5p	hsa-miR-3064-3p	hsa-miR-495-3p
hsa-miR-1305	hsa-miR-4762-5p	hsa-miR-6501-5p	hsa-miR-4775
hsa-miR-29a-3p	hsa-miR-3191-5p	hsa-miR-760	hsa-miR-374b-5p
hsa-miR-653-5p	hsa-miR-3190-3p	hsa-miR-3166	hsa-miR-1283
hsa-miR-4760-3p	hsa-miR-6732-3p	hsa-miR-4299	hsa-miR-4642
hsa-miR-513b-3p	hsa-miR-12131	hsa-miR-296-3p	hsa-miR-628-3p

© 2020. Fulzele S et al. Published online at http://www.aginganddisease.org/EN/10.14336/AD.2020.0428

SUPPLEMENTARY DATA

hsa-miR-4524b-5p	hsa-miR-5700	hsa-miR-569	hsa-miR-141-3p
hsa-miR-4524a-5p	hsa-miR-4460	hsa-miR-1200	hsa-miR-1305
hsa-miR-758-5p	hsa-miR-340-5p	hsa-miR-4765	hsa-miR-29a-3p
hsa-miR-302e	hsa-miR-4789-5p	hsa-miR-371b-3p	hsa-miR-653-5p
hsa-miR-219a-1-3p	hsa-miR-514a-5p	hsa-miR-1229-5p	hsa-miR-4760-3p
hsa-miR-6869-5p	hsa-miR-3935	hsa-miR-759	hsa-miR-513b-3p
hsa-miR-3065-3p	hsa-miR-3618	hsa-miR-34a-3p	hsa-miR-4524b-5p
hsa-miR-194-5p	hsa-miR-181a-2-3p	hsa-miR-1237-3p	hsa-miR-4524a-5p
hsa-miR-1279	hsa-miR-12119	hsa-miR-5582-5p	hsa-miR-758-5p
hsa-miR-3182	hsa-miR-432-5p	hsa-miR-5703	hsa-miR-302e
hsa-miR-3065-5p	hsa-miR-549a-3p	hsa-miR-4434	hsa-miR-219a-1-3p
hsa-miR-152-3p	hsa-miR-4652-3p	hsa-miR-8057	hsa-miR-6869-5p
hsa-miR-148b-3p	hsa-miR-6766-3p	hsa-miR-548q	hsa-miR-200a-3p
hsa-miR-148a-3p	hsa-miR-4782-3p	hsa-miR-5197-5p	hsa-miR-3065-3p
hsa-miR-451b	hsa-miR-219a-5p	hsa-miR-876-5p	hsa-miR-194-5p
hsa-miR-3120-3p	hsa-miR-1264	hsa-miR-12122	hsa-miR-1279
hsa-miR-510-3p	hsa-miR-616-3p	hsa-miR-6868-3p	hsa-miR-3182
hsa-miR-548p	hsa-miR-302a-5p	hsa-miR-134-5p	hsa-miR-3065-5p
hsa-miR-335-3p	hsa-miR-583	hsa-miR-3158-5p	hsa-miR-152-3p
hsa-miR-3613-5p	hsa-miR-324-3p	hsa-miR-4745-5p	hsa-miR-148b-3p
hsa-miR-330-3p	hsa-miR-3148	hsa-miR-5094	hsa-miR-148a-3p
hsa-miR-6515-3p	hsa-miR-4660	hsa-miR-526b-5p	hsa-miR-451b
hsa-miR-33a-3p	hsa-miR-4495	hsa-miR-3059-5p	hsa-miR-3120-3p
hsa-miR-3149	hsa-miR-8073	hsa-miR-143-5p	hsa-miR-510-3p
hsa-miR-298	hsa-miR-221-5p	hsa-miR-549a-5p	hsa-miR-548p
hsa-miR-125b-2-3p	hsa-miR-195-3p	hsa-miR-5571-3p	hsa-miR-6509-5p
hsa-miR-216b-3p	hsa-miR-16-2-3p	hsa-miR-648	hsa-miR-335-3p
hsa-miR-3185	hsa-miR-942-5p	hsa-miR-758-3p	hsa-miR-3613-5p
hsa-miR-6844	hsa-miR-499a-5p	hsa-miR-7705	hsa-miR-330-3p
hsa-miR-3912-5p	hsa-miR-561-5p	hsa-miR-670-3p	hsa-miR-6515-3p
hsa-miR-411-5p	hsa-miR-1178-5p	hsa-miR-556-3p	hsa-miR-33a-3p
hsa-miR-374a-3p	hsa-miR-1181	hsa-miR-6867-5p	hsa-miR-3149
hsa-miR-4282	hsa-miR-198	hsa-miR-1909-5p	hsa-miR-3671
hsa-miR-8075	hsa-miR-643	hsa-miR-320a-5p	hsa-miR-298
hsa-miR-603	hsa-miR-924	hsa-miR-3156-5p	hsa-miR-125b-2-3p
hsa-miR-4659b-3p	hsa-miR-4469	hsa-miR-4530	hsa-miR-216b-3p
hsa-miR-4659a-3p	hsa-miR-12114	hsa-miR-3913-3p	hsa-miR-3185
hsa-miR-4724-3p	hsa-miR-29b-2-5p	hsa-miR-1267	hsa-miR-6844
hsa-miR-624-5p	hsa-miR-2113	hsa-miR-4773	hsa-miR-3912-5p
hsa-miR-548aw	hsa-miR-664a-3p	hsa-miR-3118	hsa-miR-411-5p
hsa-miR-375-3p	hsa-miR-4743-3p	hsa-miR-9902	hsa-miR-374a-3p

© 2020. Fulzele S et al. Published online at http://www.aginganddisease.org/EN/10.14336/AD.2020.0428

SUPPLEMENTARY DATA

hsa-miR-497-3p	hsa-miR-9985	hsa-miR-6893-5p	hsa-miR-4282
hsa-miR-3123	hsa-miR-27b-3p	hsa-miR-6808-5p	hsa-miR-8075
hsa-miR-548l	hsa-miR-27a-3p	hsa-miR-425-5p	hsa-miR-603
hsa-miR-4742-3p	hsa-miR-7854-3p	hsa-miR-335-5p	hsa-miR-4659b-3p
hsa-miR-2052	hsa-miR-6797-3p	hsa-miR-1273h-3p	hsa-miR-4659a-3p
hsa-miR-4262	hsa-miR-4445-5p	hsa-miR-6818-3p	hsa-miR-4724-3p
hsa-miR-493-5p	hsa-miR-126-5p	hsa-miR-3974	hsa-miR-624-5p
hsa-miR-222-5p	hsa-miR-4666b	hsa-miR-6886-5p	hsa-miR-548aw
hsa-miR-802	hsa-miR-552-3p	hsa-miR-892b	hsa-miR-375-3p
hsa-miR-412-3p	hsa-miR-6832-5p	hsa-miR-575	hsa-miR-497-3p
hsa-miR-8054	hsa-miR-203a-5p	hsa-miR-4771	hsa-miR-3123
hsa-miR-548k	hsa-miR-6792-3p	hsa-miR-4780	hsa-miR-548l
hsa-miR-548av-5p	hsa-miR-6802-5p	hsa-miR-10226	hsa-miR-4742-3p
hsa-miR-548j-3p	hsa-miR-548ar-3p	hsa-miR-3128	hsa-miR-2052
hsa-miR-5003-5p	hsa-miR-208a-5p	hsa-miR-150-5p	hsa-miR-4262
hsa-miR-676-5p	hsa-miR-1277-5p	hsa-miR-10397-5p	hsa-miR-4504
hsa-miR-675-3p	hsa-miR-6837-3p	hsa-miR-6777-3p	hsa-miR-493-5p
hsa-miR-548t-3p	hsa-miR-208b-5p	hsa-miR-6882-5p	hsa-miR-222-5p
hsa-miR-548ap-3p	hsa-miR-103a-1-5p	hsa-miR-1287-5p	hsa-miR-802
hsa-miR-548aa	hsa-miR-548az-3p	hsa-miR-7855-5p	hsa-miR-582-3p
hsa-miR-3692-3p	hsa-miR-6529-5p	hsa-miR-6831-5p	hsa-miR-6124
hsa-miR-4753-3p	hsa-miR-103a-2-5p	hsa-miR-509-3p	hsa-miR-412-3p
hsa-miR-585-5p	hsa-miR-6510-5p	hsa-miR-6779-3p	hsa-miR-8054
hsa-miR-107	hsa-miR-4506	hsa-miR-636	hsa-miR-548k
hsa-miR-103a-3p	hsa-miR-216a-3p	hsa-miR-940	hsa-miR-548av-5p
hsa-miR-4766-3p	hsa-miR-128-3p	hsa-miR-586	hsa-miR-548j-3p
hsa-miR-6754-3p	hsa-miR-6806-5p	hsa-miR-1208	hsa-miR-5003-5p
hsa-miR-664b-3p	hsa-miR-4727-3p	hsa-miR-3927-3p	hsa-miR-676-5p
hsa-miR-579-3p	hsa-miR-5692c	hsa-miR-5193	hsa-miR-570-5p
hsa-miR-548t-5p	hsa-miR-5692b	hsa-miR-6791-3p	hsa-miR-548ba
hsa-miR-548az-5p	hsa-miR-218-2-3p	hsa-miR-6872-3p	hsa-miR-548ai
hsa-miR-3159	hsa-miR-551b-5p	hsa-miR-6882-3p	hsa-miR-548ag
hsa-miR-4778-5p	hsa-miR-7856-5p	hsa-miR-676-3p	hsa-miR-4527
hsa-miR-449b-3p	hsa-miR-3977	hsa-miR-3167	hsa-miR-675-3p
hsa-miR-4501	hsa-miR-589-5p	hsa-miR-384	hsa-miR-5684
hsa-miR-7978	hsa-miR-6761-5p	hsa-miR-519e-3p	hsa-miR-548t-3p
hsa-miR-548aq-3p	hsa-miR-618	hsa-miR-4500	hsa-miR-548ap-3p
hsa-miR-548am-3p	hsa-miR-4756-3p	hsa-miR-4486	hsa-miR-548aa
hsa-miR-548ah-3p	hsa-miR-3126-5p	hsa-miR-515-3p	hsa-miR-3692-3p
hsa-miR-548ae-3p	hsa-miR-524-5p	hsa-miR-6830-5p	hsa-miR-4753-3p
hsa-miR-548g-3p	hsa-miR-520d-5p	hsa-miR-33b-3p	hsa-miR-585-5p

© 2020. Fulzele S et al. Published online at http://www.aginganddisease.org/EN/10.14336/AD.2020.0428

SUPPLEMENTARY DATA

hsa-miR-5682	hsa-miR-4275	hsa-miR-4718	hsa-miR-107
hsa-miR-548x-5p	hsa-miR-584-5p	hsa-miR-4671-3p	hsa-miR-103a-3p
hsa-miR-548g-5p	hsa-miR-548f-3p	hsa-miR-490-5p	hsa-miR-4766-3p
hsa-miR-548aj-5p	hsa-miR-548e-3p	hsa-miR-4272	hsa-miR-6754-3p
hsa-miR-101-3p	hsa-miR-548bc	hsa-miR-130b-5p	hsa-miR-664b-3p
hsa-miR-7157-5p	hsa-miR-548a-3p	hsa-miR-3188	hsa-miR-579-3p
hsa-miR-4452	hsa-miR-323a-3p	hsa-miR-7162-5p	hsa-miR-548t-5p
hsa-miR-139-5p	hsa-miR-7844-5p	hsa-miR-516b-3p	hsa-miR-548az-5p
hsa-miR-2054	hsa-miR-4260	hsa-miR-516a-3p	hsa-miR-3159
hsa-miR-4703-5p	hsa-miR-5192	hsa-miR-6762-3p	hsa-miR-4778-5p
hsa-miR-3925-5p	hsa-miR-4307	hsa-miR-2116-5p	hsa-miR-449b-3p
hsa-miR-513c-3p	hsa-miR-380-5p	hsa-miR-3670	hsa-miR-4501
hsa-miR-513a-3p	hsa-miR-520a-5p	hsa-miR-596	hsa-miR-7978
hsa-miR-4717-3p	hsa-miR-1324	hsa-miR-5591-3p	hsa-miR-548n
hsa-miR-4735-5p	hsa-miR-655-5p	hsa-miR-5589-3p	hsa-miR-548aq-3p
hsa-miR-3674	hsa-miR-3620-3p	hsa-miR-377-3p	hsa-miR-548am-3p
hsa-miR-1256	hsa-miR-5580-3p	hsa-miR-4318	hsa-miR-548ah-3p
hsa-miR-548x-3p	hsa-miR-4742-5p	hsa-miR-127-5p	hsa-miR-548ae-3p
hsa-miR-548aj-3p	hsa-miR-543	hsa-miR-4793-5p	hsa-miR-548g-3p
hsa-miR-503-5p	hsa-miR-31-5p	hsa-miR-5699-5p	hsa-miR-6503-5p
hsa-miR-936	hsa-miR-15b-3p	hsa-miR-500b-5p	hsa-miR-5682
hsa-miR-153-3p	hsa-miR-3134	hsa-miR-362-5p	hsa-miR-548x-5p
hsa-miR-5696	hsa-miR-642b-3p	hsa-miR-2467-3p	hsa-miR-548g-5p
hsa-miR-197-3p	hsa-miR-642a-3p	hsa-miR-6780a-3p	hsa-miR-548aj-5p
hsa-miR-548e-5p	hsa-miR-148a-5p	hsa-miR-5692a	hsa-miR-101-3p
hsa-miR-548f-5p	hsa-miR-3928-3p	hsa-miR-7111-3p	hsa-miR-7157-5p
hsa-miR-6875-3p	hsa-miR-4509	hsa-miR-593-3p	hsa-miR-4452
hsa-miR-584-3p	hsa-miR-218-1-3p	hsa-miR-938	hsa-miR-139-5p
hsa-miR-103b	hsa-miR-4668-3p	hsa-miR-6126	hsa-miR-4744
hsa-miR-6866-5p	hsa-miR-3163	hsa-miR-5586-3p	hsa-miR-2054
hsa-miR-4528	hsa-miR-5702	hsa-miR-4499	hsa-miR-4703-5p
hsa-miR-885-5p	hsa-miR-548v	hsa-miR-152-5p	hsa-miR-3925-5p
hsa-miR-634	hsa-miR-6771-3p	hsa-miR-3619-3p	hsa-miR-513c-3p
hsa-miR-4677-3p	hsa-miR-29a-5p	hsa-miR-205-5p	hsa-miR-513a-3p
hsa-miR-183-3p	hsa-miR-1197	hsa-miR-6768-5p	hsa-miR-4717-3p
hsa-miR-5195-3p	hsa-miR-10b-3p	hsa-miR-378g	hsa-miR-4735-5p
hsa-miR-145-5p	hsa-miR-4670-3p	hsa-miR-196a-3p	hsa-miR-3674
hsa-miR-548at-5p	hsa-miR-607	hsa-miR-1290	hsa-miR-1256
hsa-miR-624-3p	hsa-miR-12120	hsa-miR-199b-5p	hsa-miR-142-5p
hsa-miR-3529-3p	hsa-miR-6892-5p	hsa-miR-199a-5p	hsa-miR-6800-5p
hsa-miR-3973	hsa-miR-4303	hsa-miR-665	hsa-miR-577

© 2020. Fulzele S et al. Published online at http://www.aginganddisease.org/EN/10.14336/AD.2020.0428

SUPPLEMENTARY DATA

hsa-miR-7108-5p	hsa-miR-2681-5p	hsa-miR-548c-3p	hsa-miR-548x-3p
hsa-miR-6728-3p	hsa-miR-541-5p	hsa-miR-433-3p	hsa-miR-548aj-3p
hsa-miR-595	hsa-miR-212-3p	hsa-miR-1323	hsa-miR-605-5p
hsa-miR-561-3p	hsa-miR-132-3p	hsa-miR-5587-5p	hsa-miR-503-5p
hsa-miR-3941	hsa-miR-6076	hsa-miR-4764-3p	hsa-miR-936
hsa-miR-3680-3p	hsa-miR-659-3p	hsa-miR-544b	hsa-miR-5590-3p
hsa-miR-7-2-3p	hsa-miR-6801-5p	hsa-miR-4639-3p	hsa-miR-153-3p
hsa-miR-7-1-3p	hsa-miR-10393-3p	hsa-miR-365b-3p	hsa-miR-5696
hsa-miR-28-3p	hsa-miR-4280	hsa-miR-365a-3p	hsa-miR-552-5p
hsa-miR-6507-3p	hsa-miR-138-2-3p	hsa-miR-4438	hsa-miR-197-3p
hsa-miR-346	hsa-miR-1-5p	hsa-miR-6855-5p	hsa-miR-6805-3p
hsa-miR-3942-5p	hsa-miR-3681-3p	hsa-miR-93-5p	hsa-miR-5691
hsa-miR-421	hsa-miR-934	hsa-miR-519d-3p	hsa-miR-548e-5p
hsa-miR-627-5p	hsa-miR-3121-3p	hsa-miR-20b-5p	hsa-miR-548f-5p
hsa-miR-8068	hsa-miR-4305	hsa-miR-17-5p	hsa-miR-6875-3p
hsa-let-7c-3p	hsa-miR-3646	hsa-miR-617	hsa-miR-584-3p
hsa-miR-641	hsa-miR-4766-5p	hsa-miR-4697-3p	hsa-miR-485-3p
hsa-miR-3617-5p	hsa-miR-668-3p	hsa-miR-6879-3p	hsa-miR-103b
hsa-miR-519c-3p	hsa-miR-563	hsa-miR-193a-5p	hsa-miR-6866-5p
hsa-miR-519b-3p	hsa-miR-9-3p	hsa-miR-182-3p	hsa-miR-4528
hsa-miR-519a-3p	hsa-miR-562	hsa-miR-3651	hsa-miR-539-3p
hsa-miR-3606-3p	hsa-miR-6134	hsa-miR-4676-5p	hsa-miR-8063
hsa-miR-4531	hsa-miR-518c-5p	hsa-miR-20a-5p	hsa-miR-885-5p
hsa-miR-100-3p	hsa-miR-1911-3p	hsa-miR-106b-5p	hsa-miR-634
hsa-miR-29b-1-5p	hsa-miR-4650-3p	hsa-miR-6765-3p	hsa-miR-4677-3p
hsa-miR-380-3p	hsa-miR-4463	hsa-miR-4736	hsa-miR-183-3p
hsa-miR-571	hsa-miR-1252-3p	hsa-miR-1225-5p	hsa-miR-5195-3p
hsa-miR-6776-3p	hsa-miR-217-3p	hsa-miR-7847-3p	hsa-miR-145-5p
hsa-miR-3910	hsa-miR-3978	hsa-miR-106a-5p	hsa-miR-548at-5p
hsa-miR-6790-5p	hsa-miR-4661-5p	hsa-miR-5196-5p	hsa-miR-624-3p
hsa-miR-4658	hsa-miR-125a-3p	hsa-miR-4747-5p	hsa-miR-302d-3p
hsa-miR-1297	hsa-miR-525-5p	hsa-miR-1253	hsa-miR-302c-3p
hsa-miR-1468-3p	hsa-miR-4638-3p	hsa-miR-203b-3p	hsa-miR-302b-3p
hsa-miR-646	hsa-miR-3918	hsa-miR-526b-3p	hsa-miR-302a-3p
hsa-miR-1184	hsa-miR-31-3p	hsa-miR-6813-3p	hsa-miR-3529-3p
hsa-miR-5047	hsa-miR-154-3p	hsa-miR-6858-3p	hsa-miR-3973
hsa-miR-580-3p	hsa-miR-4432	hsa-miR-7156-3p	hsa-miR-7108-5p
hsa-miR-4801	hsa-miR-4712-3p	hsa-miR-4643	hsa-miR-6728-3p
hsa-miR-297	hsa-miR-4699-3p	hsa-miR-4457	hsa-miR-325
hsa-miR-514b-3p	hsa-miR-570-3p	hsa-miR-548o-3p	hsa-miR-595
hsa-miR-514a-3p	hsa-miR-422a	hsa-miR-4256	hsa-miR-561-3p

© 2020. Fulzele S et al. Published online at http://www.aginganddisease.org/EN/10.14336/AD.2020.0428

SUPPLEMENTARY DATA

hsa-miR-3658	hsa-miR-378i	hsa-miR-11181-5p	hsa-miR-3941
hsa-miR-499b-3p	hsa-miR-378h	hsa-miR-6730-3p	hsa-miR-4483
hsa-miR-499a-3p	hsa-miR-378f	hsa-miR-217-5p	hsa-miR-3680-3p
hsa-miR-8081	hsa-miR-378e	hsa-miR-4257	hsa-miR-7-2-3p
hsa-miR-4694-5p	hsa-miR-378d	hsa-miR-133a-5p	hsa-miR-7-1-3p
hsa-miR-4709-5p	hsa-miR-378c	hsa-miR-3157-5p	hsa-miR-28-3p
hsa-miR-7112-3p	hsa-miR-378b	hsa-miR-6807-3p	hsa-miR-6507-3p
hsa-miR-8485	hsa-miR-378a-3p	hsa-miR-147b-5p	hsa-miR-20a-3p
hsa-miR-4786-3p	hsa-miR-4673	hsa-miR-5708	hsa-miR-346
hsa-miR-3143	hsa-miR-4760-5p	hsa-miR-135a-2-3p	hsa-miR-3942-5p
hsa-miR-1250-3p	hsa-miR-6516-5p	hsa-miR-138-1-3p	hsa-miR-421
hsa-miR-1231	hsa-miR-6877-3p	hsa-miR-1263	hsa-miR-627-5p
hsa-miR-4731-3p	hsa-miR-190a-3p	hsa-miR-548av-3p	hsa-miR-647
hsa-miR-651-5p	hsa-miR-1269b	hsa-miR-5586-5p	hsa-miR-8068
hsa-miR-4659b-5p	hsa-miR-1269a	hsa-miR-3922-5p	hsa-let-7c-3p
hsa-miR-521	hsa-miR-6079	hsa-miR-4999-5p	hsa-miR-641
hsa-miR-4799-5p	hsa-miR-151a-3p	hsa-miR-6781-3p	hsa-miR-3617-5p
hsa-miR-494-3p	hsa-miR-5697	hsa-miR-190b-5p	hsa-miR-5087
hsa-miR-219a-2-3p	hsa-miR-3184-3p	hsa-miR-190a-5p	hsa-miR-519c-3p
hsa-miR-3650	hsa-miR-3688-3p	hsa-miR-6800-3p	hsa-miR-519b-3p
hsa-miR-376b-3p	hsa-miR-4496	hsa-miR-4436b-5p	hsa-miR-519a-3p
hsa-miR-376a-3p	hsa-miR-206	hsa-miR-4709-3p	hsa-miR-3606-3p
hsa-miR-144-3p	hsa-miR-1-3p	hsa-miR-1272	hsa-miR-4531
hsa-miR-302d-5p	hsa-miR-6750-3p	hsa-miR-6504-3p	hsa-miR-100-3p
hsa-miR-302b-5p	hsa-miR-15a-3p	hsa-miR-6859-5p	hsa-miR-6863
hsa-miR-5582-3p	hsa-miR-4645-5p	hsa-miR-11399	hsa-miR-23c
hsa-miR-6820-3p	hsa-miR-5683	hsa-miR-6829-5p	hsa-miR-23b-3p
hsa-miR-3164	hsa-miR-6505-3p	hsa-miR-330-5p	hsa-miR-23a-3p
hsa-miR-3688-5p	hsa-miR-6819-3p	hsa-miR-8064	hsa-miR-30e-3p
hsa-miR-130a-5p	hsa-miR-3609	hsa-miR-4251	hsa-miR-30d-3p
hsa-miR-95-5p	hsa-miR-219b-5p	hsa-miR-223-3p	hsa-miR-30a-3p
hsa-miR-644a	hsa-miR-5572	hsa-miR-6720-5p	hsa-miR-29b-1-5p
hsa-miR-6507-5p	hsa-miR-616-5p	hsa-miR-6512-3p	hsa-miR-380-3p
hsa-miR-892c-3p	hsa-miR-373-5p	hsa-miR-144-5p	hsa-miR-122b-5p
hsa-miR-4676-3p	hsa-miR-371b-5p	hsa-miR-18b-5p	hsa-miR-571
hsa-miR-452-5p	hsa-miR-7850-5p	hsa-miR-18a-5p	hsa-miR-6776-3p
hsa-miR-4635	hsa-miR-5706	hsa-miR-7150	hsa-miR-6719-3p
hsa-miR-4711-5p	hsa-miR-4782-5p	hsa-miR-4722-5p	hsa-miR-3910
hsa-miR-4659a-5p	hsa-miR-1288-3p	hsa-miR-185-3p	hsa-miR-6790-5p
hsa-miR-219b-3p	hsa-miR-613	hsa-miR-136-3p	hsa-miR-4658
hsa-miR-8055	hsa-miR-593-5p	hsa-miR-2682-3p	hsa-miR-1297

© 2020. Fulzele S et al. Published online at http://www.aginganddisease.org/EN/10.14336/AD.2020.0428

SUPPLEMENTARY DATA

hsa-miR-891a-3p	hsa-miR-659-5p	hsa-miR-4647	hsa-miR-1468-3p
hsa-miR-1236-3p	hsa-miR-6759-5p	hsa-miR-6807-5p	hsa-miR-4733-3p
hsa-miR-361-5p	hsa-miR-8061	hsa-miR-10399-5p	hsa-miR-646
hsa-miR-6818-5p	hsa-miR-4529-5p	hsa-miR-3689f	hsa-miR-1184
hsa-miR-147a	hsa-miR-620	hsa-miR-3689e	hsa-miR-4714-5p
hsa-miR-3920	hsa-miR-1270	hsa-miR-3689b-5p	hsa-miR-145-3p
hsa-miR-1301-3p	hsa-miR-215-3p	hsa-miR-3689a-5p	hsa-miR-5047
hsa-miR-4429	hsa-let-7f-1-3p	hsa-miR-7975	hsa-miR-6875-5p
hsa-miR-320d	hsa-miR-424-3p	NA	hsa-miR-580-3p
hsa-miR-320c	hsa-miR-187-5p	NA	hsa-miR-4801
hsa-miR-320b	hsa-miR-4451	NA	hsa-miR-297
hsa-miR-320a-3p	hsa-miR-10523-5p	NA	hsa-miR-514b-3p
hsa-miR-4772-3p	hsa-miR-223-5p	NA	hsa-miR-514a-3p
hsa-miR-4325	hsa-miR-548ah-5p	NA	hsa-miR-3658
hsa-miR-374b-3p	hsa-miR-5579-5p	NA	hsa-miR-499b-3p
hsa-miR-4758-5p	hsa-miR-6071	NA	hsa-miR-499a-3p
hsa-miR-3177-5p	hsa-miR-6730-5p	NA	hsa-miR-8081
hsa-miR-382-5p	hsa-miR-3924	NA	hsa-miR-4694-5p
hsa-miR-4679	hsa-miR-6766-5p	NA	hsa-miR-4709-5p
hsa-miR-522-3p	hsa-miR-6756-5p	NA	hsa-miR-7112-3p
hsa-miR-224-3p	hsa-miR-1183	NA	hsa-miR-8485
hsa-miR-299-5p	hsa-miR-4316	NA	hsa-miR-4786-3p
hsa-miR-3121-5p	hsa-miR-6852-3p	NA	hsa-miR-520d-3p
hsa-miR-576-5p	hsa-miR-654-3p	NA	hsa-miR-520a-3p
hsa-miR-4795-3p	hsa-miR-4534	NA	hsa-miR-1293
hsa-miR-218-5p	hsa-miR-487a-3p	NA	hsa-miR-3143
hsa-miR-6809-3p	hsa-miR-548ao-3p	NA	hsa-miR-1250-3p
hsa-miR-32-3p	hsa-miR-6715b-3p	NA	hsa-miR-1231
hsa-miR-181d-5p	hsa-miR-1266-3p	NA	hsa-miR-4731-3p
hsa-miR-6755-3p	hsa-miR-591	NA	hsa-miR-651-5p
hsa-miR-597-3p	hsa-miR-150-3p	NA	hsa-miR-4719
hsa-miR-1257	hsa-miR-6874-5p	NA	hsa-miR-411-3p
hsa-miR-494-5p	hsa-miR-875-5p	NA	hsa-miR-379-3p
hsa-miR-2053	NA	NA	hsa-miR-4659b-5p
hsa-miR-3664-5p	NA	NA	hsa-miR-521
hsa-miR-3662	NA	NA	hsa-miR-4799-5p
hsa-miR-6165	NA	NA	hsa-miR-494-3p
hsa-miR-4263	NA	NA	hsa-miR-4774-5p
hsa-miR-6502-3p	NA	NA	hsa-miR-219a-2-3p
hsa-miR-587	NA	NA	hsa-miR-3650
hsa-miR-597-5p	NA	NA	hsa-miR-376b-3p

hsa-miR-203a-3p	NA		NA		hsa-miR-376a-3p
hsa-miR-1179	NA		NA		hsa-miR-144-3p
hsa-miR-3934-5p	NA		NA		hsa-miR-302d-5p
hsa-miR-498-3p	NA		NA		hsa-miR-302b-5p
hsa-miR-627-3p	NA		NA		hsa-miR-508-3p
hsa-miR-511-5p	NA		NA		hsa-miR-5582-3p
hsa-miR-181c-5p	NA		NA		hsa-miR-3614-3p
hsa-miR-181b-5p	NA		NA		hsa-miR-34c-3p
hsa-miR-181a-5p	NA		NA		hsa-miR-6820-3p
hsa-miR-20b-3p	NA		NA		hsa-miR-3164
hsa-miR-590-3p	NA		NA		hsa-miR-3688-5p
hsa-miR-649	NA		NA		hsa-miR-130a-5p
hsa-miR-10527-5p	NA		NA		hsa-miR-95-5p
hsa-miR-3130-3p	NA		NA		hsa-miR-644a
hsa-miR-1238-5p	NA		NA		hsa-miR-6828-5p
hsa-miR-410-5p	NA		NA		hsa-miR-6507-5p
hsa-miR-323b-5p	NA		NA		hsa-miR-520e-3p
hsa-miR-6811-3p	NA		NA		hsa-miR-520c-3p
hsa-miR-5571-5p	NA		NA		hsa-miR-520b-3p
hsa-miR-1244	NA		NA		hsa-miR-373-3p
hsa-miR-3911	NA		NA		hsa-miR-372-3p
hsa-miR-520h	NA		NA		hsa-miR-4708-5p
hsa-miR-520g-3p	NA		NA		hsa-miR-3606-5p
hsa-miR-3617-3p	NA		NA		hsa-miR-892c-3p
hsa-miR-3908	NA		NA		hsa-miR-4676-3p
hsa-miR-4328	NA		NA		hsa-miR-452-5p
hsa-miR-4295	NA		NA		hsa-miR-4635
hsa-miR-3666	NA		NA		hsa-miR-4711-5p
hsa-miR-301b-3p	NA		NA		hsa-miR-4659a-5p
hsa-miR-301a-3p	NA		NA		hsa-miR-219b-3p
hsa-miR-130b-3p	NA		NA		hsa-miR-8055
hsa-miR-130a-3p	NA		NA		hsa-miR-891a-3p
hsa-miR-4502	NA		NA		hsa-miR-1236-3p
hsa-miR-3129-5p	NA		NA		hsa-miR-361-5p
hsa-miR-199b-3p	NA		NA		hsa-miR-6818-5p
hsa-miR-199a-3p	NA		NA		hsa-miR-147a
hsa-miR-548an	NA		NA		hsa-miR-3920
hsa-miR-7110-3p	NA		NA		hsa-miR-1301-3p
hsa-miR-4703-3p	NA		NA		hsa-miR-4429
hsa-miR-3152-3p	NA		NA		hsa-miR-320d
hsa-miR-4803	NA		NA		hsa-miR-320c

© 2020. Fulzele S et al. Published online at http://www.aginganddisease.org/EN/10.14336/AD.2020.0428

hsa-miR-2276-3p	NA		NA		hsa-miR-320b
hsa-miR-3611	NA		NA		hsa-miR-320a-3p
hsa-miR-6817-5p	NA		NA		hsa-miR-4772-3p
hsa-miR-4769-3p	NA		NA		hsa-miR-4762-5p
hsa-miR-4690-5p	NA		NA		hsa-miR-4325
hsa-miR-26b-5p	NA		NA		hsa-miR-374b-3p
hsa-miR-26a-5p	NA		NA		hsa-miR-4758-5p
hsa-miR-4255	NA		NA		hsa-miR-3191-5p
hsa-miR-4738-3p	NA		NA		hsa-miR-3177-5p
hsa-miR-2355-3p	NA		NA		hsa-miR-382-5p
hsa-miR-4698	NA		NA		hsa-miR-4679
hsa-miR-192-3p	NA		NA		hsa-miR-522-3p
hsa-miR-33b-5p	NA		NA		hsa-miR-224-3p
hsa-miR-33a-5p	NA		NA		hsa-miR-3190-3p
hsa-miR-498-5p	NA		NA		hsa-miR-6732-3p
hsa-miR-767-5p	NA		NA		hsa-miR-12131
hsa-miR-454-3p	NA		NA		hsa-miR-299-5p
hsa-miR-4468	NA		NA		hsa-miR-3121-5p
hsa-miR-6739-3p	NA		NA		hsa-miR-5700
hsa-miR-567	NA		NA		hsa-miR-4460
hsa-miR-122-5p	NA		NA		hsa-miR-340-5p
hsa-miR-136-5p	NA		NA		hsa-miR-576-5p
hsa-miR-181c-3p	NA		NA		hsa-miR-4789-5p
hsa-miR-452-3p	NA		NA		hsa-miR-4795-3p
hsa-miR-10395-5p	NA		NA		hsa-miR-218-5p
hsa-miR-4291	NA		NA		hsa-miR-6809-3p
hsa-miR-6733-3p	NA		NA		hsa-miR-32-3p
hsa-miR-507	NA		NA		hsa-miR-514a-5p
hsa-miR-10522-5p	NA		NA		hsa-miR-3935
hsa-miR-548b-3p	NA		NA		hsa-miR-181d-5p
hsa-miR-4465	NA		NA		hsa-miR-6755-3p
hsa-miR-5585-3p	NA		NA		hsa-miR-597-3p
hsa-miR-4691-5p	NA		NA		hsa-miR-1257
hsa-miR-4729	NA		NA		hsa-miR-494-5p
hsa-miR-573	NA		NA		hsa-miR-2053
hsa-miR-338-3p	NA		NA		hsa-miR-3618
hsa-miR-4264	NA		NA		hsa-miR-181a-2-3p
hsa-miR-5197-3p	NA		NA		hsa-miR-3664-5p
hsa-miR-511-3p	NA		NA		hsa-miR-3662
hsa-miR-7161-5p	NA		NA		hsa-miR-6165
hsa-miR-500a-5p	NA		NA		hsa-miR-12119

© 2020. Fulzele S et al. Published online at http://www.aginganddisease.org/EN/10.14336/AD.2020.0428

SUPPLEMENTARY DATA

hsa-miR-142-3p	NA		NA		hsa-miR-4263
hsa-miR-4682	NA		NA		hsa-miR-6502-3p
hsa-miR-1227-3p	NA		NA		hsa-miR-432-5p
hsa-miR-362-3p	NA		NA		hsa-miR-549a-3p
hsa-miR-5010-3p	NA		NA		hsa-miR-587
hsa-miR-4536-5p	NA		NA		hsa-miR-4652-3p
hsa-miR-557	NA		NA		hsa-miR-597-5p
hsa-miR-1322	NA		NA		hsa-miR-6766-3p
hsa-miR-542-3p	NA		NA		hsa-miR-4782-3p
hsa-miR-3189-3p	NA		NA		hsa-miR-219a-5p
hsa-miR-944	NA		NA		hsa-miR-203a-3p
hsa-miR-6796-3p	NA		NA		hsa-miR-1179
hsa-miR-513b-5p	NA		NA		hsa-miR-1264
hsa-miR-5680	NA		NA		hsa-miR-3934-5p
hsa-miR-574-5p	NA		NA		hsa-miR-616-3p
hsa-miR-6833-5p	NA		NA		hsa-miR-302a-5p
hsa-miR-519d-5p	NA		NA		hsa-miR-498-3p
hsa-miR-367-5p	NA		NA		hsa-miR-627-3p
hsa-miR-4999-3p	NA		NA		hsa-miR-511-5p
hsa-miR-12117	NA		NA		hsa-miR-583
hsa-miR-4797-3p	NA		NA		hsa-miR-324-3p
hsa-miR-4677-5p	NA		NA		hsa-miR-181c-5p
hsa-miR-383-3p	NA		NA		hsa-miR-181b-5p
hsa-miR-506-3p	NA		NA		hsa-miR-181a-5p
hsa-miR-124-3p	NA		NA		hsa-miR-20b-3p
hsa-miR-6716-5p	NA		NA		hsa-miR-590-3p
hsa-miR-16-1-3p	NA		NA		hsa-miR-649
hsa-miR-323a-5p	NA		NA		hsa-miR-3148
hsa-miR-7702	NA		NA		hsa-miR-10527-5p
hsa-miR-19b-2-5p	NA		NA		hsa-miR-3130-3p
hsa-miR-19b-1-5p	NA		NA		hsa-miR-1238-5p
hsa-miR-19a-5p	NA		NA		hsa-miR-410-5p
hsa-miR-6513-3p	NA		NA		hsa-miR-323b-5p
hsa-miR-4711-3p	NA		NA		hsa-miR-6811-3p
hsa-miR-578	NA		NA		hsa-miR-5571-5p
hsa-miR-3622b-3p	NA		NA		hsa-miR-4660
hsa-miR-3622a-3p	NA		NA		hsa-miR-1244
hsa-miR-548ay-3p	NA		NA		hsa-miR-4495
hsa-miR-548at-3p	NA		NA		hsa-miR-3911
hsa-miR-548u	NA		NA		hsa-miR-520h
hsa-let-7g-3p	NA		NA		hsa-miR-520g-3p

© 2020. Fulzele S et al. Published online at http://www.aginganddisease.org/EN/10.14336/AD.2020.0428

SUPPLEMENTARY DATA

hsa-let-7a-2-3p	NA		NA		hsa-miR-3617-3p
hsa-miR-3616-5p	NA		NA		hsa-miR-8073
hsa-miR-532-5p	NA		NA		hsa-miR-221-5p
hsa-miR-4312	NA		NA		hsa-miR-195-3p
hsa-miR-1265	NA		NA		hsa-miR-16-2-3p
hsa-miR-6826-5p	NA		NA		hsa-miR-3908
hsa-miR-581	NA		NA		hsa-miR-4328
hsa-miR-4716-5p	NA		NA		hsa-miR-942-5p
hsa-miR-544a	NA		NA		hsa-miR-4295
hsa-miR-4748	NA		NA		hsa-miR-3666
hsa-miR-4289	NA		NA		hsa-miR-301b-3p
hsa-miR-5690	NA		NA		hsa-miR-301a-3p
hsa-miR-4464	NA		NA		hsa-miR-130b-3p
hsa-miR-495-5p	NA		NA		hsa-miR-130a-3p
hsa-miR-153-5p	NA		NA		hsa-miR-4502
hsa-miR-376c-3p	NA		NA		hsa-miR-3129-5p
hsa-miR-876-3p	NA		NA		hsa-miR-199b-3p
hsa-miR-6782-3p	NA		NA		hsa-miR-199a-3p
hsa-miR-519e-5p	NA		NA		hsa-miR-548an
hsa-miR-515-5p	NA		NA		hsa-miR-499a-5p
hsa-miR-3619-5p	NA		NA		hsa-miR-7110-3p
hsa-miR-3161	NA		NA		hsa-miR-561-5p
hsa-miR-5011-3p	NA		NA		hsa-miR-4703-3p
hsa-miR-329-3p	NA		NA		hsa-miR-1178-5p
hsa-miR-450b-5p	NA		NA		hsa-miR-3152-3p
hsa-miR-4267	NA		NA		hsa-miR-1181
hsa-miR-6884-3p	NA		NA		hsa-miR-4803
hsa-miR-6847-3p	NA		NA		hsa-miR-2276-3p
hsa-miR-4633-5p	NA		NA		hsa-miR-3611
hsa-miR-4793-3p	NA		NA		hsa-miR-6817-5p
hsa-miR-140-3p	NA		NA		hsa-miR-4769-3p
hsa-miR-592	NA		NA		hsa-miR-198
hsa-miR-770-5p	NA		NA		hsa-miR-4690-5p
hsa-miR-4712-5p	NA		NA		hsa-miR-643
hsa-miR-4694-3p	NA		NA		hsa-miR-924
hsa-miR-3180-5p	NA		NA		hsa-miR-26b-5p
hsa-miR-922	NA		NA		hsa-miR-26a-5p
hsa-miR-520f-5p	NA		NA		hsa-miR-4255
hsa-miR-937-5p	NA		NA		hsa-miR-4469
hsa-miR-4426	NA		NA		hsa-miR-12114
hsa-miR-3923	NA		NA		hsa-miR-4738-3p

© 2020. Fulzele S et al. Published online at http://www.aginganddisease.org/EN/10.14336/AD.2020.0428

SUPPLEMENTARY DATA

hsa-miR-656-5p	NA		NA		hsa-miR-29b-2-5p
hsa-miR-329-5p	NA		NA		hsa-miR-2355-3p
hsa-miR-369-3p	NA		NA		hsa-miR-4698
hsa-miR-4680-3p	NA		NA		hsa-miR-192-3p
hsa-miR-3972	NA		NA		hsa-miR-33b-5p
hsa-miR-1202	NA		NA		hsa-miR-33a-5p
hsa-miR-5693	NA		NA		hsa-miR-498-5p
hsa-miR-7109-3p	NA		NA		hsa-miR-767-5p
hsa-miR-891b	NA		NA		hsa-miR-2113
hsa-miR-4456	NA		NA		hsa-miR-664a-3p
hsa-miR-4789-3p	NA		NA		hsa-miR-4743-3p
hsa-miR-3976	NA		NA		hsa-miR-454-3p
hsa-miR-338-5p	NA		NA		hsa-miR-4468
hsa-miR-4662a-3p	NA		NA		hsa-miR-9985
hsa-miR-4705	NA		NA		hsa-miR-27b-3p
hsa-miR-3659	NA		NA		hsa-miR-27a-3p
hsa-miR-12135	NA		NA		hsa-miR-6739-3p
hsa-miR-7153-3p	NA		NA		hsa-miR-567
hsa-miR-214-3p	NA		NA		hsa-miR-122-5p
hsa-miR-548ax	NA		NA		hsa-miR-7854-3p
hsa-miR-548ao-5p	NA		NA		hsa-miR-6797-3p
hsa-miR-3675-3p	NA		NA		hsa-miR-136-5p
hsa-miR-214-5p	NA		NA		hsa-miR-181c-3p
hsa-miR-4678	NA		NA		hsa-miR-452-3p
hsa-miR-520e-5p	NA		NA		hsa-miR-10395-5p
hsa-miR-140-5p	NA		NA		hsa-miR-4291
hsa-miR-5003-3p	NA		NA		hsa-miR-6733-3p
hsa-miR-582-5p	NA		NA		hsa-miR-507
hsa-miR-6740-3p	NA		NA		hsa-miR-4445-5p
hsa-miR-12129	NA		NA		hsa-miR-126-5p
hsa-miR-1276	NA		NA		hsa-miR-4666b
hsa-miR-4691-3p	NA		NA		hsa-miR-552-3p
hsa-miR-761	NA		NA		hsa-miR-6832-5p
hsa-miR-5695	NA		NA		hsa-miR-203a-5p
hsa-miR-9851-3p	NA		NA		hsa-miR-6792-3p
hsa-miR-4503	NA		NA		hsa-miR-10522-5p
hsa-miR-6841-5p	NA		NA		hsa-miR-6802-5p
hsa-miR-5002-5p	NA		NA		hsa-miR-548b-3p
hsa-miR-6792-5p	NA		NA		hsa-miR-4465
hsa-miR-4776-3p	NA		NA		hsa-miR-5585-3p
hsa-miR-1915-5p	NA		NA		hsa-miR-4691-5p

© 2020. Fulzele S et al. Published online at http://www.aginganddisease.org/EN/10.14336/AD.2020.0428

hsa-miR-6514-3p		NA		NA		hsa-miR-4729
hsa-miR-8062		NA		NA		hsa-miR-548ar-3p
hsa-miR-3919		NA		NA		hsa-miR-573
hsa-miR-4511		NA		NA		hsa-miR-338-3p
hsa-miR-6849-3p		NA		NA		hsa-miR-208a-5p
hsa-miR-12127		NA		NA		hsa-miR-4264
hsa-miR-655-3p		NA		NA		hsa-miR-1277-5p
hsa-miR-374c-5p		NA		NA		hsa-miR-6837-3p
hsa-miR-555		NA		NA		hsa-miR-5197-3p
hsa-miR-4477a		NA		NA		hsa-miR-208b-5p
hsa-miR-4261		NA		NA		hsa-miR-511-3p
hsa-miR-196b-5p		NA		NA		hsa-miR-103a-1-5p
hsa-miR-196a-5p		NA		NA		hsa-miR-7161-5p
hsa-miR-892a		NA		NA		hsa-miR-500a-5p
hsa-miR-372-5p		NA		NA		hsa-miR-142-3p
hsa-miR-4693-5p		NA		NA		hsa-miR-4682
hsa-miR-12136		NA		NA		hsa-miR-548az-3p
hsa-miR-193b-3p		NA		NA		hsa-miR-6529-5p
hsa-miR-193a-3p		NA		NA		hsa-miR-1227-3p
hsa-miR-22-5p		NA		NA		hsa-miR-103a-2-5p
hsa-miR-502-5p		NA		NA		hsa-miR-6510-5p
hsa-miR-4685-3p		NA		NA		hsa-miR-362-3p
hsa-miR-4287		NA		NA		hsa-miR-5010-3p
hsa-miR-3942-3p		NA		NA		hsa-miR-4536-5p
hsa-miR-7849-3p		NA		NA		hsa-miR-4506
hsa-miR-331-5p		NA		NA		hsa-miR-557
hsa-miR-155-5p		NA		NA		hsa-miR-1322
hsa-miR-182-5p		NA		NA		hsa-miR-216a-3p
hsa-miR-4662a-5p		NA		NA		hsa-miR-128-3p
hsa-miR-4645-3p		NA		NA		hsa-miR-6806-5p
						hsa-miR-4727-3p
						hsa-miR-542-3p
						hsa-miR-5692c
						hsa-miR-5692b
						hsa-miR-3189-3p
						hsa-miR-944
						hsa-miR-6796-3p
						hsa-miR-218-2-3p
						hsa-miR-513b-5p
						hsa-miR-5680
						hsa-miR-551b-5p

© 2020. Fulzele S et al. Published online at http://www.aginganddisease.org/EN/10.14336/AD.2020.0428

SUPPLEMENTARY DATA

						hsa-miR-7856-5p
						hsa-miR-574-5p
						hsa-miR-6833-5p
						hsa-miR-3977
						hsa-miR-519d-5p
						hsa-miR-589-5p
						hsa-miR-367-5p
						hsa-miR-4999-3p
						hsa-miR-6761-5p
						hsa-miR-618
						hsa-miR-12117
						hsa-miR-4756-3p
						hsa-miR-4797-3p
						hsa-miR-3126-5p
						hsa-miR-524-5p
						hsa-miR-520d-5p
						hsa-miR-4275
						hsa-miR-4677-5p
						hsa-miR-584-5p
						hsa-miR-548f-3p
						hsa-miR-548e-3p
						hsa-miR-548bc
						hsa-miR-383-3p
						hsa-miR-506-3p
						hsa-miR-124-3p
						hsa-miR-548a-3p
						hsa-miR-6716-5p
						hsa-miR-323a-3p
						hsa-miR-16-1-3p
						hsa-miR-323a-5p
						hsa-miR-7844-5p
						hsa-miR-7702
						hsa-miR-19b-2-5p
						hsa-miR-19b-1-5p
						hsa-miR-19a-5p
						hsa-miR-6513-3p
						hsa-miR-4260
						hsa-miR-4711-3p
						hsa-miR-5192
						hsa-miR-578
						hsa-miR-3622b-3p

© 2020. Fulzele S et al. Published online at http://www.aginganddisease.org/EN/10.14336/AD.2020.0428

						hsa-miR-3622a-3p
						hsa-miR-4307
						hsa-miR-380-5p
						hsa-miR-520a-5p
						hsa-miR-1324
						hsa-miR-548ay-3p
						hsa-miR-548at-3p
						hsa-miR-655-5p
						hsa-miR-3620-3p
						hsa-miR-5580-3p
						hsa-miR-4742-5p
						hsa-miR-543
						hsa-miR-548u
						hsa-miR-31-5p
						hsa-let-7g-3p
						hsa-let-7a-2-3p
						hsa-miR-3616-5p
						hsa-miR-532-5p
						hsa-miR-4312
						hsa-miR-15b-3p
						hsa-miR-1265
						hsa-miR-6826-5p
						hsa-miR-3134
						hsa-miR-642b-3p
						hsa-miR-642a-3p
						hsa-miR-148a-5p
						hsa-miR-581
						hsa-miR-3928-3p
						hsa-miR-4716-5p
						hsa-miR-544a
						hsa-miR-4748
						hsa-miR-4509
						hsa-miR-4289
						hsa-miR-218-1-3p
						hsa-miR-5690
						hsa-miR-4668-3p
						hsa-miR-3163
						hsa-miR-5702
						hsa-miR-548v
						hsa-miR-4464
						hsa-miR-495-5p

© 2020. Fulzele S et al. Published online at http://www.aginganddisease.org/EN/10.14336/AD.2020.0428

						hsa-miR-6771-3p
						hsa-miR-153-5p
						hsa-miR-29a-5p
						hsa-miR-376c-3p
						hsa-miR-876-3p
						hsa-miR-6782-3p
						hsa-miR-519e-5p
						hsa-miR-515-5p
						hsa-miR-1197
						hsa-miR-10b-3p
						hsa-miR-4670-3p
						hsa-miR-3619-5p
						hsa-miR-3161
						hsa-miR-5011-3p
						hsa-miR-607
						hsa-miR-329-3p
						hsa-miR-450b-5p
						hsa-miR-12120
						hsa-miR-6892-5p
						hsa-miR-4267
						hsa-miR-4303
						hsa-miR-2681-5p
						hsa-miR-6884-3p
						hsa-miR-6847-3p
						hsa-miR-4633-5p
						hsa-miR-4793-3p
						hsa-miR-140-3p
						hsa-miR-541-5p
						hsa-miR-212-3p
						hsa-miR-132-3p
						hsa-miR-592
						hsa-miR-6076
						hsa-miR-659-3p
						hsa-miR-6801-5p
						hsa-miR-10393-3p
						hsa-miR-4280
						hsa-miR-770-5p
						hsa-miR-4712-5p
						hsa-miR-4694-3p
						hsa-miR-138-2-3p
						hsa-miR-1-5p

© 2020. Fulzele S et al. Published online at http://www.aginganddisease.org/EN/10.14336/AD.2020.0428

						hsa-miR-3681-3p
						hsa-miR-934
						hsa-miR-3180-5p
						hsa-miR-3121-3p
						hsa-miR-922
						hsa-miR-4305
						hsa-miR-520f-5p
						hsa-miR-3646
						hsa-miR-937-5p
						hsa-miR-4426
						hsa-miR-4766-5p
						hsa-miR-668-3p
						hsa-miR-563
						hsa-miR-3923
						hsa-miR-656-5p
						hsa-miR-329-5p
						hsa-miR-369-3p
						hsa-miR-9-3p
						hsa-miR-4680-3p
						hsa-miR-562
						hsa-miR-6134
						hsa-miR-3972
						hsa-miR-518c-5p
						hsa-miR-1202
						hsa-miR-1911-3p
						hsa-miR-4650-3p
						hsa-miR-5693
						hsa-miR-7109-3p
						hsa-miR-891b
						hsa-miR-4463
						hsa-miR-4456
						hsa-miR-4789-3p
						hsa-miR-1252-3p
						hsa-miR-217-3p
						hsa-miR-3976
						hsa-miR-3978
						hsa-miR-4661-5p
						hsa-miR-338-5p
						hsa-miR-125a-3p
						hsa-miR-525-5p
						hsa-miR-4662a-3p

© 2020. Fulzele S et al. Published online at http://www.aginganddisease.org/EN/10.14336/AD.2020.0428

SUPPLEMENTARY DATA

						hsa-miR-4638-3p
						hsa-miR-3918
						hsa-miR-31-3p
						hsa-miR-4705
						hsa-miR-3659
						hsa-miR-154-3p
						hsa-miR-12135
						hsa-miR-7153-3p
						hsa-miR-4432
						hsa-miR-214-3p
						hsa-miR-548ax
						hsa-miR-548ao-5p
						hsa-miR-4712-3p
						hsa-miR-4699-3p
						hsa-miR-570-3p
						hsa-miR-3675-3p
						hsa-miR-422a
						hsa-miR-378i
						hsa-miR-378h
						hsa-miR-378f
						hsa-miR-378e
						hsa-miR-378d
						hsa-miR-378c
						hsa-miR-378b
						hsa-miR-378a-3p
						hsa-miR-4673
						hsa-miR-214-5p
						hsa-miR-4678
						hsa-miR-4760-5p
						hsa-miR-520e-5p
						hsa-miR-6516-5p
						hsa-miR-6877-3p
						hsa-miR-140-5p
						hsa-miR-5003-3p
						hsa-miR-582-5p
						hsa-miR-6740-3p
						hsa-miR-190a-3p
						hsa-miR-1269b
						hsa-miR-1269a
						hsa-miR-6079
						hsa-miR-12129

© 2020. Fulzele S et al. Published online at http://www.aginganddisease.org/EN/10.14336/AD.2020.0428

						hsa-miR-151a-3p
						hsa-miR-5697
						hsa-miR-1276
						hsa-miR-4691-3p
						hsa-miR-3184-3p
						hsa-miR-761
						hsa-miR-5695
						hsa-miR-9851-3p
						hsa-miR-3688-3p
						hsa-miR-4503
						hsa-miR-6841-5p
						hsa-miR-4496
						hsa-miR-206
						hsa-miR-1-3p
						hsa-miR-5002-5p
						hsa-miR-6792-5p
						hsa-miR-6750-3p
						hsa-miR-15a-3p
						hsa-miR-4776-3p
						hsa-miR-1915-5p
						hsa-miR-6514-3p
						hsa-miR-8062
						hsa-miR-3919
						hsa-miR-4645-5p
						hsa-miR-5683
						hsa-miR-6505-3p
						hsa-miR-6819-3p
						hsa-miR-4511
						hsa-miR-6849-3p
						hsa-miR-3609
						hsa-miR-219b-5p
						hsa-miR-5572
						hsa-miR-616-5p
						hsa-miR-373-5p
						hsa-miR-371b-5p
						hsa-miR-12127
						hsa-miR-7850-5p
						hsa-miR-655-3p
						hsa-miR-374c-5p
						hsa-miR-555
						hsa-miR-5706

© 2020. Fulzele S et al. Published online at http://www.aginganddisease.org/EN/10.14336/AD.2020.0428

SUPPLEMENTARY DATA

						hsa-miR-4782-5p
						hsa-miR-1288-3p
						hsa-miR-613
						hsa-miR-593-5p
						hsa-miR-4477a
						hsa-miR-659-5p
						hsa-miR-6759-5p
						hsa-miR-4261
						hsa-miR-8061
						hsa-miR-196b-5p
						hsa-miR-196a-5p
						hsa-miR-4529-5p
						hsa-miR-620
						hsa-miR-1270
						hsa-miR-215-3p
						hsa-miR-892a
						hsa-let-7f-1-3p
						hsa-miR-424-3p
						hsa-miR-187-5p
						hsa-miR-4451
						hsa-miR-10523-5p
						hsa-miR-372-5p
						hsa-miR-4693-5p
						hsa-miR-12136
						hsa-miR-223-5p
						hsa-miR-548ah-5p
						hsa-miR-5579-5p
						hsa-miR-193b-3p
						hsa-miR-193a-3p
						hsa-miR-22-5p
						hsa-miR-6071
						hsa-miR-502-5p
						hsa-miR-6730-5p
						hsa-miR-4685-3p
						hsa-miR-4287
						hsa-miR-3942-3p
						hsa-miR-3924
						hsa-miR-7849-3p
						hsa-miR-6766-5p
						hsa-miR-6756-5p
						hsa-miR-331-5p

© 2020. Fulzele S et al. Published online at http://www.aginganddisease.org/EN/10.14336/AD.2020.0428

						hsa-miR-155-5p
						hsa-miR-1183
						hsa-miR-4316
						hsa-miR-182-5p
						hsa-miR-6852-3p
						hsa-miR-654-3p
						hsa-miR-4534
						hsa-miR-487a-3p
						hsa-miR-4662a-5p
						hsa-miR-548ao-3p
						hsa-miR-6715b-3p
						hsa-miR-1266-3p
						hsa-miR-4645-3p
						hsa-miR-591
						hsa-miR-150-3p
						hsa-miR-6874-5p
						hsa-miR-875-5p

© 2020. Fulzele S et al. Published online at http://www.aginganddisease.org/EN/10.14336/AD.2020.0428
